# Regulatory factor X 7 limits Myc activity during B cell activation and suppresses Myc-dependent lymphomagenesis

**DOI:** 10.1038/s41590-026-02526-2

**Published:** 2026-06-01

**Authors:** Berenice A. Fischer, Hanif J. Khameneh, Jessica Guerra, Alessandro Zenobi, Jasmine Ciorciari, Irene Buzzago, Maria Serena Merli, Mohammed Said, Pedro M. O. Ventura, Alessandra Pfister, Lara Mattei, Daniele Cavalli, Francesca Molinari, Andrea Rinaldi, Simone G. Moro, Ivo Kwee, Diego Morone, Simone Mosole, David Jarrossay, Chiara Montanino, Luca Simonelli, Luca Varani, Egle Radice, Marcus Thelen, Clelia Di Serio, Eleonora Calabretta, Margaret A. Shipp, Kostantyn Dreval, Ryan Morin, David W. Scott, Federico Jauk, Milo Frattini, Jessica Barizzi, Luciano Cascione, Francesco Bertoni, Davide F. Robbiani, Davide Rossi, Greta Guarda

**Affiliations:** 1 Institute for Research in Biomedicine, Università della Svizzera italiana, Bellinzona, Switzerland; 2 Cantonal Institute of Pathology Ente Ospedaliero Cantonale (EOC), Locarno, Switzerland; 3 Institute of Oncology Research, Università della Svizzera italiana, Bellinzona, Switzerland; 4 BigOmics Analytics SA, Lugano, Switzerland; 5 University Centre of Statistics in the Biomedical Sciences (CUSSB), Milan, Italy; 6 Vita-Salute San Raffaele University, Milan, Italy; 7 Biomedical Faculty, Università della Svizzera Italiana, Lugano, Switzerland; 8 Clinical Trial Unit (CTU), EOC, Locarno, Switzerland; 9 Department of Medical Oncology, Dana-Farber Cancer Institute, Boston, MA USA; 10 Department of Molecular Biology and Biochemistry, Simon Fraser University, Burnaby, British Columbia Canada; 11 Centre for Lymphoid Cancer, BC Cancer, Vancouver, British Columbia Canada; 12 Canada’s Michael Smith Genome Sciences Centre at BC Cancer, Vancouver, British Columbia Canada; 13 Division of Hematology, Oncology Institute of Southern Switzerland, EOC, Bellinzona, Switzerland

**Keywords:** Lymphoma, B cells

## Abstract

Regulatory factor X 7 (*RFX7*) nonsense mutations have been found in different human B cell malignancies. We therefore set out to study the role of RFX7 in B cell activation and lymphomagenesis. Here we show that *RFX7* truncations cause loss-of-function and dominant-negative effects. Moreover, low *RFX7* mRNA levels correlate with worse diffuse large B cell lymphoma prognosis. Accordingly, *Rfx7* deletion in B cells accelerates pathogenesis in mouse Bcl6- and p53-loss-driven B cell lymphoma models. Rfx7-deficient B cells exhibit increased Myc activity and enhanced germinal center B cell and plasmablast responses. These alterations are reverted by Myc haploinsufficiency, which provides partial protection from nonsymptomatic *p53*^−/−^*Rfx7*^−/−^ B cell lymphoma, but does not prevent detrimental Myc deregulation in aggressive disease. Deletion of *Aicda*, which favors genomic alterations in activated B cells, limits lymphoma development in the *p53*^−/−^*Rfx7*^−/−^ double-hit mouse model. These results indicate that Rfx7 represses B cell activation, Myc activity, and Myc- and activation-induced cytidine deaminase (AID)-dependent pro-lymphomagenic processes.

## Abstract (web summary)

Guarda and colleagues investigate the role of the transcription factor RFX7 in B cell activation and lymphomagenesis. The findings indicate that Rfx7 regulates germinal center B cell, plasmablast generation and malignant transformation by restricting Myc activity.

## Main

RFX transcription factors are characterized by a conserved winged helix DNA-binding domain and are involved mostly in the regulation of cilia and immunity^1^. Knowledge about RFX7 has only recently started to emerge. A role for this transcription factor in ciliogenesis was reported in *Xenopus laevis.* Supporting a developmental function of Rfx7, knockout mice were born at sub-Mendelian ratios^2,3^. Moreover, we demonstrated that conditional deletion of Rfx7 in the hematopoietic system resulted in reduced natural killer (NK) cell numbers. In these lymphocytes, Rfx7 controlled the expression of genes limiting metabolic and proliferative pathways, including regulators of mechanistic target of rapamycin complex 1 (mTORC1)^3^. Accordingly, Rfx7-deficient NK cells presented augmented metabolic rates and haploinsufficiency of *Raptor*—the key molecule of the mTORC1 complex—partially normalizing the NK cell phenotype^3^.

Extending these observations to cancer, a human osteosarcoma cell line knockout for RFX7 was found recently to exhibit increased mTORC1 activity, strengthening the view that this transcription factor ‘brakes’ cellular metabolic pathways^3,4^. Further analyses revealed that RFX7 controls the expression of genes with tumor suppressor functions, including *DDIT4*, which negatively regulates mTORC1 activity^3–5^. Notably, genome-wide insertional mutagenesis studies supported a tumor suppressor role for Rfx7 also in B lymphocytes^6–8^.

B cells are highly prone to transformation due to processes essential to produce antibodies, including B cell receptor (BCR) assembly, class switch recombination and somatic hypermutation, the latter taking place predominantly within germinal centers (GCs)^9^. *RFX7* mutations have been identified in 3–5% of diffuse large B cell lymphomas (DLBCLs) and in 13–15% of the Epstein–Barr-virus-negative Burkitt lymphomas (BLs)^10–16^. Moreover, a polymorphism near the *RFX7* locus correlates with chronic lymphocytic leukemia, where *RFX7* mutations have been associated to patient prognosis in the unmutated subset^17,18^. These data indicate that RFX7 is relevant for a range of blood cancers originating from activated B cells.

Since the role and relevance of RFX7 in B cells as well as the mechanisms underlying its function in B cell lymphomagenesis remain unknown, we set out to address these questions. We found that RFX7 controls the transcriptional activity of the proto-oncogene MYC in mouse B cells and a human DLBCL line. In agreement with the importance of Myc for B cell activation, Rfx7 limits the expansion of GC B cells and plasmablasts (PBs) following immunization^19–21^. Rfx7 delays lymphoma development driven by Bcl6 or p53-loss, and we identified a role for Myc and activation-induced cytidine deaminase (AID), the enzyme responsible for somatic hypermutation and class switch recombination^22^, in accelerating lymphomagenesis driven by the concomitant loss of Rfx7 and p53. Notably, low *RFX7* transcript abundance correlates with worse DLBCL prognosis. Moreover, nonsense mutations are common *RFX7* alterations in lymphoid malignancies; we found that truncated RFX7 forms lead to loss of transcription factor function, while acting as dominant-negative inhibitors of wild-type (WT) RFX7. These data provide a mechanistic understanding of how *RFX7* alterations contribute to hematologic malignancies.

## Results

### Rfx7 delays development of Bcl6-driven lymphoma

**Extended Data Fig. 1:**
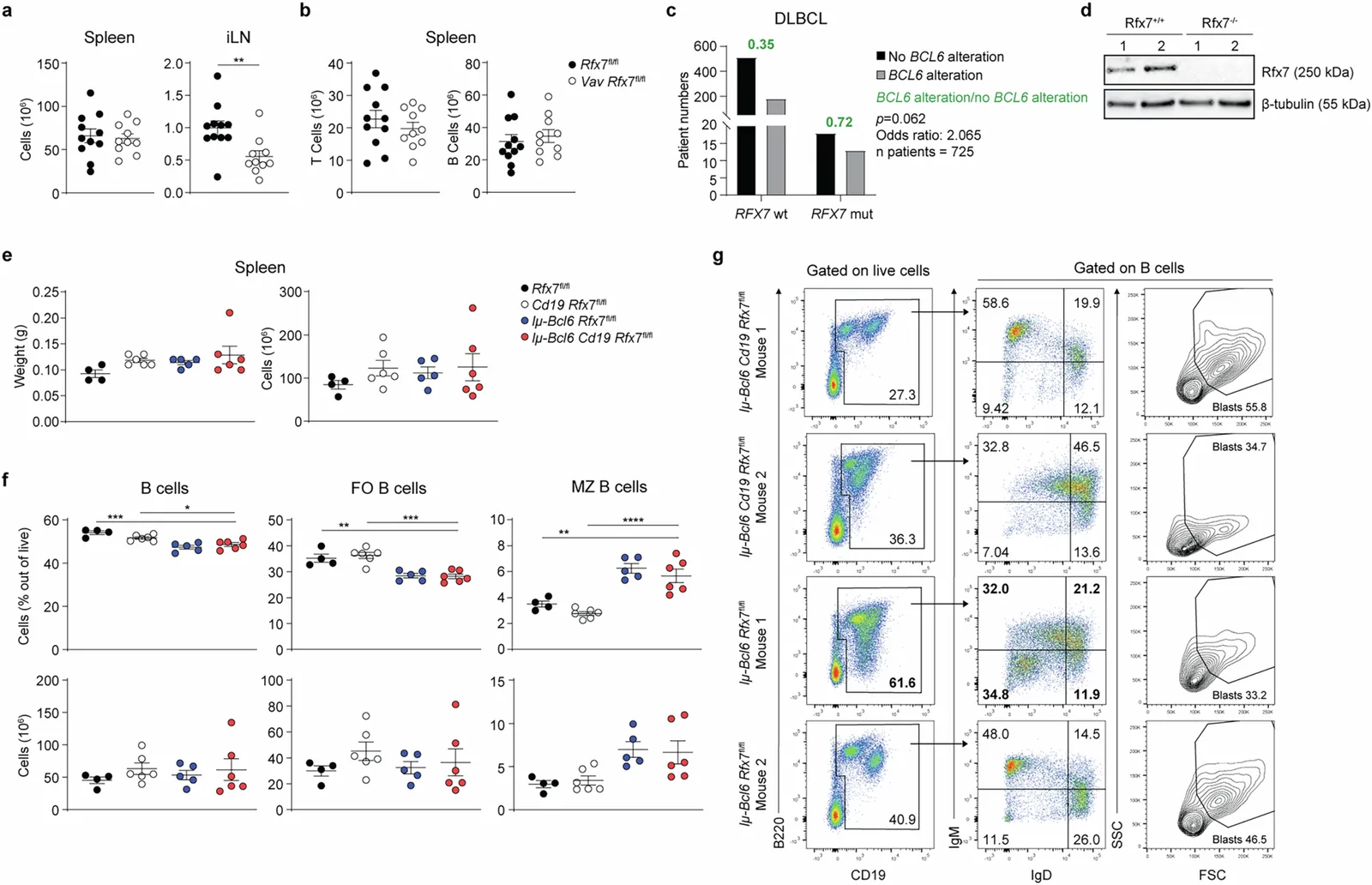
Lymphoid compartment of different genetically modified mice. **a** and **b**) Analysis of 12 to 13-month-old *VavRfx7*^fl/fl^ and *Rfx7*^fl/fl^ mice. Graphs depict cellularity of spleen and inguinal lymph nodes (iLNs) (**a**), and the number of splenic T cells (CD3^+^) and B cells (CD19^+^B220^+^) from these mice (**b**). **c**) The number and ratio of DLBCL patients^16,53,54^ with and without *BCL6* alterations are shown according to *RFX7* mutation status (related to Supplementary Table 3); Fisher’s exact test (two-sided); odds ratio (OR, 95% CI). **d**) Lysates from splenic B cells isolated from *Cd19Rfx7*^wt/wt^ and *Cd19Rfx7*^fl/fl^ mice were blotted and probed for Rfx7 and β-tubulin as loading control. **e** and **f**) Analysis of 6 to 8-week-old *Iμ-Bcl6Rfx7*^fl/fl^, *Iμ-Bcl6Cd19Rfx7*^fl/fl^, *Cd19Rfx7*^fl/fl^, and *Rfx7*^fl/fl^ mice; spleen weight and cellularity is depicted (**e**). Percentage and number of B cells (CD19^+^B220^+^), FO B cells (CD19^+^B220^+^CD23^+^), and MZ B cells (CD19^+^B220^+^CD21^+^) are shown (**f**). **g**) Examples of flow cytometry plots of B cells and their expression of IgM, IgD, and blasting profiles in spleens of 10 to 13-month- *Iμ-Bcl6Cd19Rfx7*^fl/fl^ and *Iμ-Bcl6Rfx7*^fl/fl^ mice. Results represent mean±s.e.m. of n = 10 *VavRfx7*^fl/fl^ and n = 10–11 *Rfx7*^fl/fl^ (**a** and **b**) and n = 6 *Iμ-Bcl6CD19Rfx7*^fl/fl^, n = 5 *Iμ-Bcl6Rfx7*^fl/fl^, n = 6 *CD19Rfx7*^fl/fl^, and n = 4 *Rfx7*^fl/fl^ mice (**e** and **f**) and are a pool of four (**a** and **b**) or two (**e** and **f**) experiments. Statistical comparisons were performed with unpaired t-test, two-tailed, (**a** and **b**), or one-way ANOVA corrected for multiple testing using the Dunnett method comparing *Iμ-Bcl6Cd19Rfx7*^fl/fl^ to the other groups (**e** and **f**); only significant comparisons are shown (**a**, **b**, **e** and **f**). Source data

First, we asked whether deletion of Rfx7 would cause lymphoma. We analyzed 12- to 13-month-old mice lacking Rfx7 in the hematopoietic system (Cre-recombinase under the *Vav1* promoter, referred to as *VavRfx7*^fl/fl^) and did not observe increased cellularity of spleen and inguinal lymph nodes (iLNs), or splenic T and B cells as compared to their *Rfx7*^fl/fl^ counterparts (Extended Data Fig. 1a,b). Therefore, lack of Rfx7 alone does not promote lymphoproliferation in this timeframe.

**Fig. 1:**
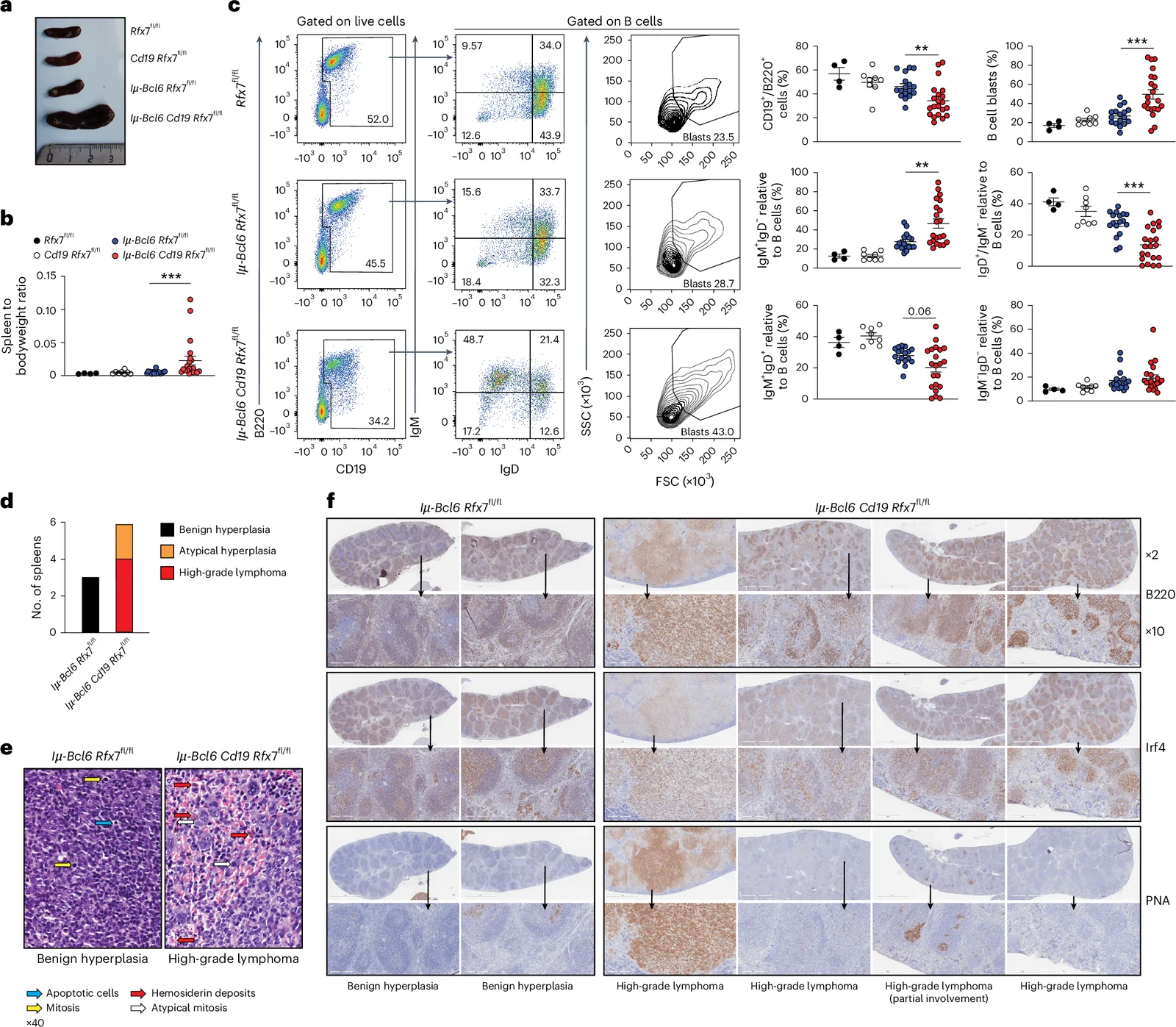
Rfx7 hinders Bcl6-driven B cell lymphoma. **a**–**f**, Analysis of 10- to 13-month-old (**a**–**c**) or 7- to 8-month-old (**d**–**f**) *Iμ-Bcl6Cd19Rfx7*^fl/fl^, *Iμ-Bcl6Rfx7*^fl/fl^, *Cd19Rfx7*^fl/fl^ and *Rfx7*^fl/fl^ mice. Representative images of spleens (**a**) and quantification of spleen weight over bodyweight (**b**) of the indicated mice are illustrated. **c**, Representative flow cytometry analyses of the spleens of aged *Iμ-Bcl6Cd19Rfx7*^fl/fl^, *Iμ-Bcl6Rfx7*^fl/fl^ and control mice and quantifications thereof. **d**, Summary of the pathological score analysis of spleen sections from *Iμ-Bcl6Cd19Rfx7*^fl/fl^ (*n* = 6) and *Iμ-Bcl6Rfx7*^fl/fl^ (*n* = 3) mice. **e**, H&E staining of the spleens of 7- to 8-month-old mice of the indicated genotype. **f**, Representative IHC images of spleen sections stained with B220, Irf-4 and PNA. Scale bars: for ×2 is 2 mm, for ×10 is 300 μm. Results represent mean ± s.e.m. of *n* = 21 *Iμ-Bcl6Cd19Rfx7*^fl/fl^, *n* = 17 *Iμ-Bcl6Rfx7*^fl/fl^, *n* = 8 *Cd19Rfx7*^fl/fl^ and *n* = 4 *Rfx7*^fl/fl^ mice; statistical comparisons between the *Iμ-Bcl6CD19Rfx7*^fl/fl^ and the *Iμ-Bcl6Rfx7*^fl/fl^ group were performed by Mann–Whitney test, two-tailed; only significant comparisons are shown (**b** and **c**); **P ≤ 0.01, ***P ≤ 0.001. FSC, forward scatter; SSC, side scatter. Source data

As *RFX7* mutations have been reported in DLBCL and are enriched numerically among cases harboring *BCL6* alterations (Extended Data Fig. 1c), we next tested whether Rfx7 contributes to tumor suppression in the *Iμ-Bcl6* mouse model, in which the proto-oncogene B cell leukemia/lymphoma 6 (Bcl6) is expressed under the immunoglobulin (Ig)M promoter mimicking a translocation common in this lymphoma^23^. We thus crossed *Iμ-Bcl6* with *Cd19CreRfx7*^fl/fl^ mice (in which Rfx7 deletion was confirmed by immunoblot; Extended Data Fig. 1d), resulting in mice with B cells expressing Bcl6 and lacking Rfx7 (hereafter called *Iμ-Bcl6Cd19Rfx7*^fl/fl^). At a young age, *Iμ-Bcl6Cd19Rfx7*^fl/fl^ mice did not present significant differences as compared to *Iμ-Bcl6Rfx7*^fl/fl^ mice, but exhibited an increased incidence of splenomegaly at 10–13 months of age (Fig. 1a,b and Extended Data Fig. 1e,f). In agreement with the original characterization of this DLBCL model^23^, spleens from *Iμ-Bcl6Cd19Rfx7*^fl/fl^ mice exhibited blastoid B cells with variably altered B cell marker expression, trends apparent—albeit less prominent—also in *Iμ-Bcl6Rfx7*^fl/fl^ mice (Fig. 1c and Extended Data Fig. 1g). Pathologic analysis of spleens from *Iμ-Bcl6Rfx7*^fl/fl^ and *Iμ-Bcl6Cd19Rfx7*^fl/fl^ mice at 7–8 months of age revealed benign hyperplasia in the former, whereas atypical hyperplasia and high-grade lymphoma were observed in the latter (Fig. 1d). *Iμ-Bcl6Rfx7*^fl/fl^ mice presented spleens with an altered splenic architecture, medium-to-large cell populations and mitotic figures (Fig. 1e). In contrast, spleens from *Iμ-Bcl6Cd19Rfx7*^fl/fl^ mice with B cell high-grade lymphoma exhibited loss of architecture, a prevailing population of large cells and typical or atypical mitotic figures; deposits of iron complexes (haemosiderin) were also observed (Fig. 1e). Complementarity-determining region 3 (CDR3) sequencing further revealed that one atypical hyperplasia presented a polyclonal repertoire, whereas the other one and the high-grade lymphomas exhibited mono- or oligo-clonal BCR repertoires with a dominant clone; mutations were identified in several of these clones (Supplementary Table 1a). This is in line with immunophenotypic analyses demonstrating expression of interferon regulatory factor 4 (Irf-4) and variable expression of peanut agglutinin (PNA), a GC B cell marker (Fig. 1f), indicative of GC or post-GC origin. Altogether, these data underscore the relevance of Rfx7 in suppressing Bcl6-driven lymphomagenesis.

### Rfx7 limits Myc activity

**Fig. 2:**
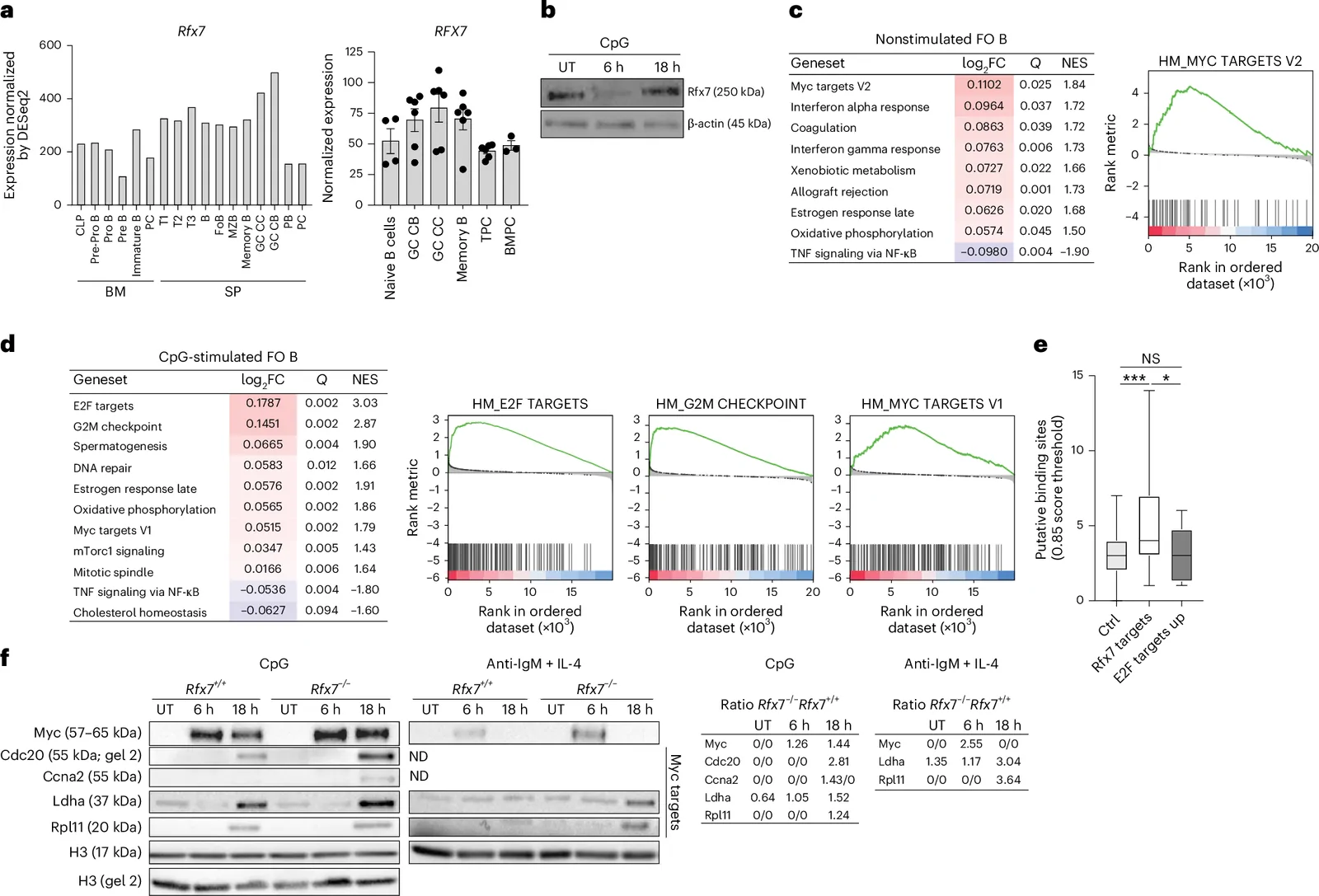
Enriched pathways in *Rfx7*^−/−^ B cells relate to Myc and proliferation. **a**, *RFX7* transcript levels in the indicated mouse (left: expression value normalized by DESeq2^65^) and human (right: normalized expression from *n* = 4 donors for naive B cells, *n* = 3 for BMPC, *n* = 6 for all other subsets^66^) B cell subsets are illustrated. **b**, Immunoblot analysis of Rfx7 expression in enriched B cells untreated (UT) or stimulated with CpG for 6 h and 18 h. β-actin is shown as loading control. **c**,**d**, Sorted splenic FO B cells (CD19^+^, B220^+^ and CD23^+^) of 6- to 12-week-old *Cd19Rfx7*^fl/fl^ (*Rfx7*^−/−^) and *Cd19Rfx7*^wt/wt^ (*Rfx7*^*+/+*^) mice were processed directly or stimulated with CpG for 18 h and RNA-seq was performed. Hallmark genesets (MSigDB; *Q* < 0.1 using fGSEA) and selected GSEA plots altered in *Rfx7*^−/−^ compared to *Rfx7*^+/+^ B cells are shown for nonstimulated (**c**) and CpG-stimulated (**d**) cells. NES, normalized enrichment score. **e**, Analysis of the promoter sequences of nonmodulated genes (*n* = 34 with log_2_FC > −0.018 and < 0.024; meta.*Q* > 0.99, serving as a control group), reported RFX7 target genes (*n* = 42, identified by ChIP^3,5^ and found to be tendentially decreased (log_2_FC < −0.2) in the current or previously published mouse RNA-seq data^3^) and the top genes (*n* = 20) of the E2F signature upregulated in CpG-activated *Rfx7*^−/−^ B cells was performed with JASPAR tool to quantify putative Rfx7 transcription factor binding sites. The box-and-whisker plots illustrate the median number of putative transcription factor binding sites identified and upper/lower quartiles (ends of the box), and maximal and minimal values (whiskers). Significance was determined with Kruskal–Wallis tests. *P ≤ 0.05, ***P ≤ 0.001. **f**, Enriched splenic B cells from *VavRfx7*^fl/fl^ (*Rfx7*^−/−^) and *Rfx7*^fl/fl^ mice (*Rfx7*^+/+^) were stimulated with CpG or anti-IgM and IL-4 and the lysates were collected 0, 6 or 18 h after stimulation and assessed by immunoblot for the indicated proteins. Histone 3 (H3) was used as a loading control and band intensity was quantified by densitometry (normalized to H3). Data are representative of at least two independent experiments. BMPC, BM plasma cell; CB, centroblast; CC, centrocyte; CLP, common lymphoid progenitor; Ctrl, control; ND, nondetected; NS, not significant; PC, plasma cell; SP, spleen; T, transitional; TPC, tonsillar PC. Source data

**Extended Data Fig. 2:**
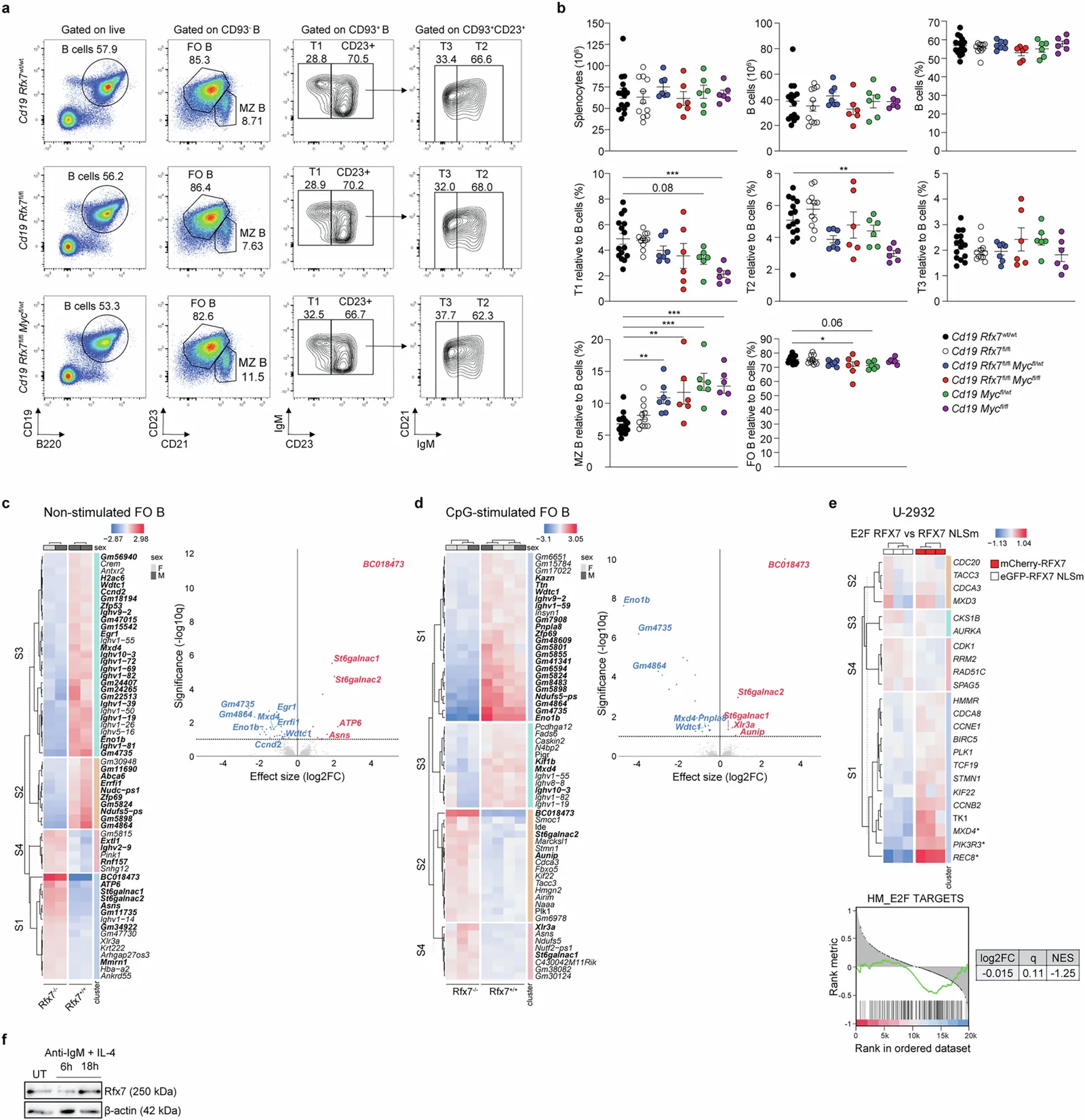
B cell compartment in mice lacking Rfx7 and Myc. **a** and **b**) Analysis of 6 to 12-week-old mice of the indicated genotypes. Representative flow cytometry plots of B cells (CD19^+^B220^+^), FO B cells (CD19^+^B220^+^CD23^+^), MZ B cells (CD19^+^B220^+^CD21^+^), T1 B cells (CD19^+^B220^+^CD93^+^CD23^−^), T2 B cells (CD19^+^B220^+^CD93^+^CD23^+^IgM^+^), and T3 B cells (CD19^+^B220^+^CD93^+^CD23^+^IgM^−^) are shown (**a**). Percentage and number of the indicated subsets are shown (**b**). Results represent mean ± s.e.m. of n = 15 *Cd19Rfx7*^wt/wt^, n = 11 *Cd19Rfx7*^fl/fl^, n = 7 *Cd19Rfx7*^fl/fl^*Myc*^fl/wt^, n = 6 *Cd19Rfx7*^fl/fl^*Myc*^fl/fl^, n = 6 *Cd19Myc*^fl/wt^, and n = 6 *Cd19Myc*^fl/fl^ mice and are a pool of three experiments. Statistical comparisons were performed with one-way ANOVA corrected for multiple testing using the Dunnett method comparing *CD19Rfx7*^wt/wt^ to the other groups; only significant results are shown (**b**). **c** and) Sorted splenic FO B cells (CD19^+^, B220^+^ and CD23^+^) of 6 to 12-week-old *Cd19Rfx7*^fl/fl^ (Rfx7^*−/−*^), and *Cd19Rfx7*^wt/wt^ (Rfx7^*+/+*^) mice were directly processed or stimulated with CpG for 18 h and RNA-sequencing (RNAseq) was performed. Hierarchical clustering of the top 60 differentially expressed genes and volcano plots showing differentially expressed genes (some of which highlighted; red upregulated and blue downregulated) for non-stimulated (**c**) and CpG-stimulated (**d**) Rfx7^*−/−*^ *vs*. Rfx7^*+/+*^cells. Statistical analysis was performed by using voom.limma test FDR < 0.1 and log2FC ≥ 0.2 and statistically significant genes are highlighted bold in the heatmaps. **e**) Heatmap created for U-2932 cells transfected with eGFP-RFX7 WT or NLS mutant (NLSm), showing the top 20 leading edge genes of the EF2 signature from the mouse RNAseq data set and three known targets (*MXD4*, *PIK3R3*, and *REC8*) indicated with an asterisk (top panel). GSEA plot shows the E2F signature comparing RFX7-WT transfected vs. RFX7-NLS mutant in U-2932 cells (down panel; not significantly altered). **f**) Immunoblot analysis of Rfx7 expression in enriched B cells untreated (UT) or stimulated with anti-IgM and IL-4 for 6 and 18 h. β-actin is shown as loading control. Source data

To understand how Rfx7 regulates lymphomagenesis, we started by characterizing its expression and relevance for normal B cells. *Rfx7* mRNA is detected in developing and splenic B cells, in which its expression at the protein level is confirmed by immunoblot (Fig. 2a,b). By analyzing *Cd19Rfx7*^fl/fl^ and control mice, we did not observe significant differences in absolute number and frequency of total, follicular (FO), marginal zone (MZ) or transitional B cells (Extended Data Fig. 2a,b). We thus investigated Rfx7-driven changes by RNA sequencing (RNA-seq) analyses of *Rfx7*^*−/−*^ and control resting FO B cells. Paired differential expression analysis revealed upregulated genes, including *Asns* and *St6galnac*, enzymes whose expression is associated with cancer progression^24–27^ (Extended Data Fig. 2c and Supplementary Table 2). Among the downregulated genes in *Rfx7*^*−/−*^ FO B cells, we found the Rfx7 targets *Mxd4* and *Ccnd2*, coding for an antagonist of Myc activity and a cyclin found to limit GC B cells entering the S-phase, respectively^3,5,28^ (Extended Data Fig. 2c). Gene set enrichment analysis (GSEA) indicated ‘Myc targets’ as the top-upregulated signature (Fig. 2c).

After a transient downregulation, Rfx7 was detected 18 h postactivation with CpG (Fig. 2b), which homogeneously stimulates B cells. RNA-seq data from *Rfx7*^−/−^ versus control FO B cells triggered with CpG for 18 h confirmed the altered expression of *Mxd4* and *St6galnac 1* and *2* (Extended Data Fig. 2d and Supplementary Table 2), and the increase in selected metabolic pathways, including ‘Myc targets’ (Fig. 2d). Notably, the pathways ‘E2F targets’ and ‘G2M checkpoint,’ linked to cell cycle, were augmented strongly in FO B cells lacking Rfx7 (Fig. 2d). As there is no evidence so far supporting a transcriptional repressive role for Rfx7^3,5^, we hypothesized the upregulation of gene expression in Rfx7-deficient B cells to be indirect. We thus compared the predicted Rfx7 binding sites in the promoter of the top genes upregulated in *Rfx7*^−/−^ activated FO B cells from the E2F signature^29^ to those of nonmodulated genes and of known RFX7 targets^3,5^. We observed a significant enrichment of putative Rfx7 binding sites in the promoters of known target genes, whereas such enrichment was absent in those from the E2F signature (Fig. 2e). Although such in silico analysis is merely indicative, the fact that the top upregulated genes of the E2F signature have not been identified as RFX7 targets^3,5,30^ and are not downregulated in DLBCL-derived U-2932 cells overexpressing RFX7 (Extended Data Fig. 2e) corroborate the hypothesis that their upregulation is an indirect effect of Rfx7 deletion.

We next stimulated control and *Rfx7*^−/−^ B lymphocytes in vitro using CpG or anti-IgM in the presence of interleukin (IL)-4, which more accurately reflects antigen-dependent activation and also stimulates Rfx7 expression by 18 h (Extended Data Fig. 2f) for immunoblot analysis. Upon activation, *Rfx7*^−/−^ B cells exhibited increased expression of Myc and its targets identified through transcriptomic analyses, such as Cyclin A2 (Ccna2), the cell division cycle protein 20 (Cdc20), lactate dehydrogenase A (Ldha)^31^ and ribosomal protein L11 (Rpl11)—another Myc-regulated protein^32^ (Fig. 2f). Collectively, these results demonstrate that Rfx7-deficient B cells engage Myc activity more efficiently than control B cells in response to BCR-dependent and -independent stimuli.

### Rfx7 limits B cell activation in vitro and in vivo

**Fig. 3:**
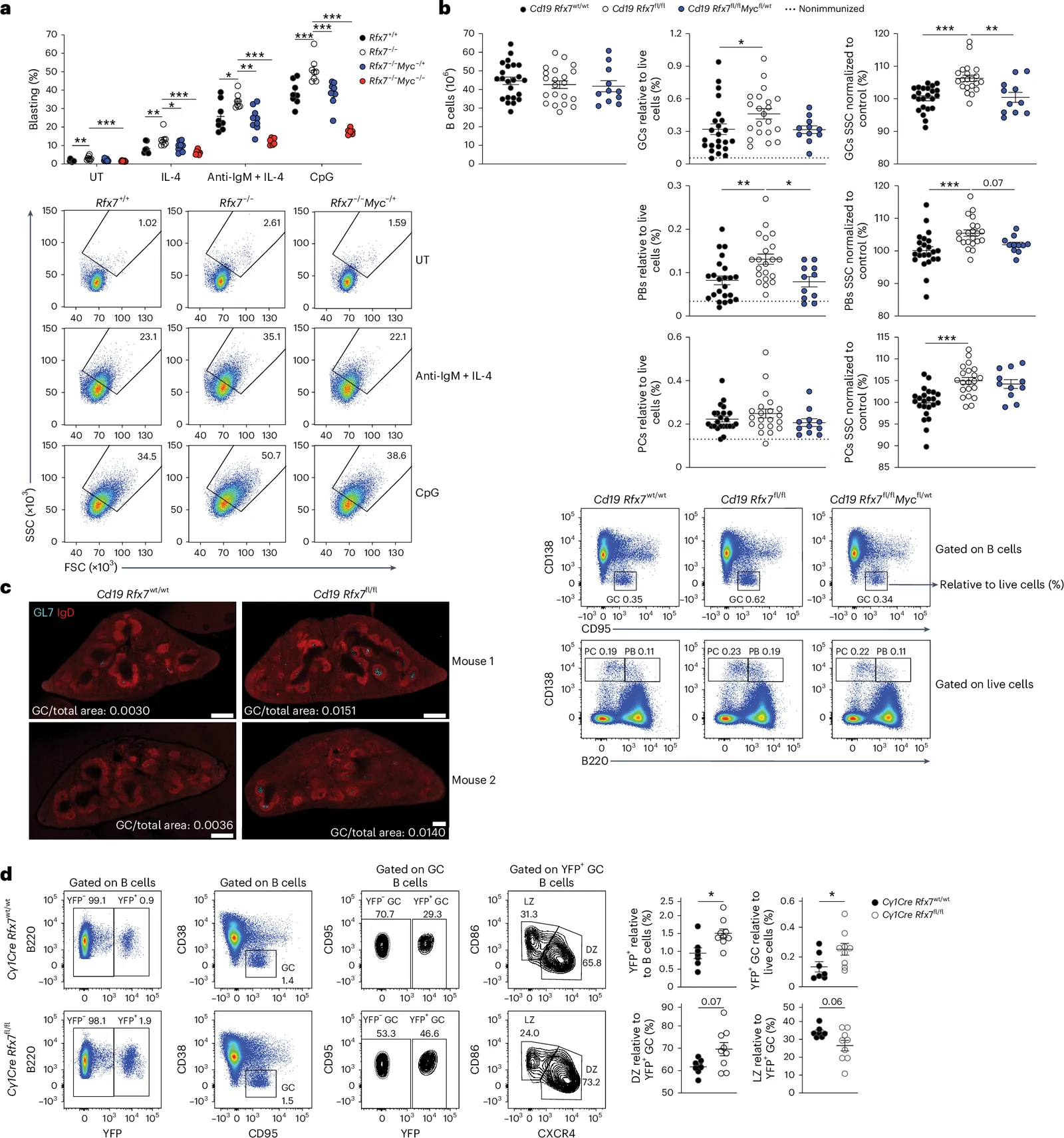
Rfx7 deletion leads to increased GC B cell and PB frequencies in vivo. **a**, Sorted splenic FO B cells from 6- to 14-week-old mice of the indicated genotypes were activated in vitro using CpG, or anti-IgM and IL-4 for 18 h or left untreated. Quantification of B cell blasts and representative flow cytometry analysis are shown. **b**,**c**, Spleens from 6- to 14-week-old mice of the indicated genotypes were analyzed 10 days after intraperitoneal immunization with NP–KLH/alum. Graphs depict total (CD19^+^, B220^+^) numbers, GC B cell (CD19^+^, B220^+^, CD38^−^, CD95^+^), PB (CD138^+^, B220^+^) and plasma cell (PC; CD138^+^, B220^−^) percentages on live cells and SSC of each subset, and flow cytometry representative plots gated as indicated in the figure (**b**). Spleen sections from immunized *Cd19Rfx7*^wt/wt^ and *Cd19Rfx7*^fl/fl^ mice were stained for IgD (red) and GL-7 (turquoise), imaged by confocal microscopy and quantified (GC/total area is indicated within the image). Scale bar, 500 µm (**c**). **d**, Graphs depict percentage of YFP^+^ B cells, YFP^+^ GC B cells, dark zone (DZ, CXCR4^hi^CD86^lo^) and light zone (LZ, CXCR4^low^CD86^hi^) YFP^+^ of GC B cells in the spleens of *Cγ1CreRfx7*^fl/fl^ and *Cγ1CreRfx7*^wt/wt^ mice 10 days after intraperitoneal immunization with NP–KLH/alum and representative flow cytometry plots. Results represent mean ± s.e.m. of *n* = 8 *Cd19Rfx7*^wt/wt^, *n* = 8 *Cd19Rfx7*^fl/fl^, *n* = 9 *Cd19Rfx7*^fl/fl^*Myc*^fl/wt^ and *n* = 7 *Cd19Rfx7*^fl/fl^*Myc*^fl/fl^ (**a**), *n* = 23 *Cd19Rfx7*^wt/wt^, *n* = 21 *Cd19Rfx7*^fl/fl^, *n* = 11 *Cd19Rfx7*^fl/fl^*Myc*^fl/wt^ (**b**) *n* = 7 *Cγ1CreRfx7*^wt/wt^ and *n* = 9 *Cγ1CreRfx7*^fl/fl^ biological replicates per group, and are a pool of two (**a**, **d**) or four (**b**) independent experiments. Dotted lines: mean of the respective readouts on nonimmunized (PBS) mice (**b**). Statistical analysis used one-way analysis of variance (ANOVA) corrected for multiple testing using the Dunnett method comparing *Cd19Rfx7*^fl/fl^ to other genotypes within each treatment condition (**a**), Kruskal–Wallis test comparing *Cd19Rfx7*^fl/fl^ to the other groups (**b**) or Mann–Whitney test, two-tailed (**d**); only significant comparisons are shown (**a**, **b**); *P ≤ 0.05, **P ≤ 0.01, ***P ≤ 0.001. Source data

**Extended Data Fig. 3:**
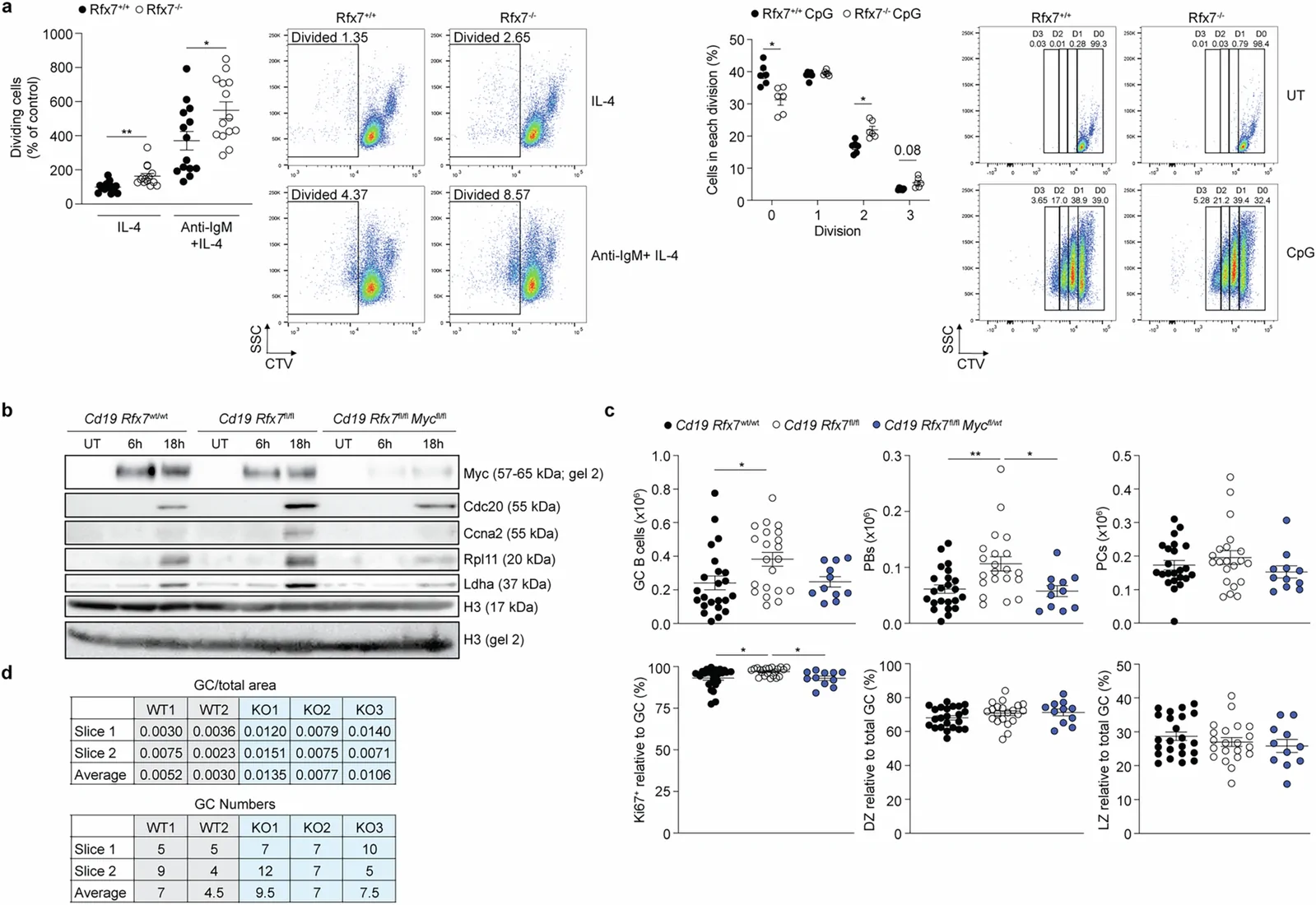
Further characterization of B cells lacking Rfx7 and Myc. **a**) Sorted CTV-labelled splenic FO B cells were stimulated with IL-4, anti-IgM+IL-4 (48 h; left panel), or with CpG or left untreated (40 h; right panel). Graphs show percentage of dividing cells (the average of the IL-4-treated Rfx7^+/+^ condition was set as 100%; left panel) and percentage of cells in each division (right panel). Representative flow cytometry plots are shown. Results represent mean±s.e.m. of n = 14 *Cd19Rfx7*^wt/wt^, n = 14 *Cd19Rfx7*^fl/fl^ and are pool of three independent experiments (left) and n = 6 *Cd19Rfx7*^wt/wt^, n = 6 *Cd19Rfx7*^fl/fl^ (right). Statistical analysis was performed comparing *Cd19Rfx7*^wt/wt^ with *Cd19Rfx7*^fl/fl^ within each condition with unpaired t-test, two-tailed; only significant comparisons are shown. **b**) Enriched splenic B cells from *Cd19Rfx7*^wt/wt^*, Cd19Rfx7*^fl/fl^, and *Cd19Rfx7*^fl/fl^*Myc*^fl/wt^ mice were stimulated for 0, 6, or 18 h with CpG and the lysates collected for immunoblot analysis for Myc, Cdc20, Ccna2, Ldha and Rpl11. Histone 3 was used as a loading control. **c**) Graphs depict numbers of GC B cells, PBs, and PCs, and percentages of Ki67^+^, dark zone (DZ; CXCR4^high^ CD86^−^), and light zone (LZ; CXCR4^low^ CD86^+^) of GC B cells in the spleens *Cd19Rfx7*^fl/fl^, *Cd19Rfx7*^wt/wt^, and *Cd19Rfx7*^fl/fl^*Myc*^fl/wt^ mice ten days after intraperitoneal immunization with NP-KLH/alum. Results represent mean±s.e.m. of n = 23 *Cd19Rfx7*^wt/wt^, n = 21 *Cd19Rfx7*^fl/fl^, n = 11 *Cd19Rfx7*^fl/fl^*Myc*^fl/wt^ mice/group and are pool of four individual experiments. Statistical analysis was performed with Kruskal-Wallis test comparing *Cd19Rfx7*^fl/fl^ to other groups; only significant comparisons are shown. **d**) Tables show quantification of GC immunofluorescence (relative to Fig. 3c), showing GC area/total area and GC numbers in two consecutive sections from n = 2 *Cd19Rfx7*^wt/wt^ (WT) and n = 3 *Cd19Rfx7*^fl/fl^ (KO) mice. Source data

Given the key role played by Myc in B cell blasting and activation^19–21^, we aimed to assess its involvement downstream of Rfx7 in vitro and in vivo. Nonstimulated *Rfx7*^−/−^ FO B cells exhibited an increased frequency of blasts, a feature that became more noticeable upon in vitro stimulation (Fig. 3a). This was accompanied by an increased proliferation, as measured by the frequency of divided cells following anti-IgM/IL-4 and of cells in each division after CpG stimulation (Extended Data Fig. 3a). We thus tested the effects of homozygous or heterozygous Myc deletion^19,33^; *Cd19Rfx7*^fl/fl^*Myc*^fl/fl^ or *Cd19Rfx7*^fl/fl^*Myc*^fl/wt^—as well as *Cd19Myc*^fl/fl^ and *Cd19Myc*^fl/wt^—mice presented normal spleen cellularity, B cell percentage and number, with increased MZ and decreased—or tendentially decreased—FO and early transitional B cells (Extended Data Fig. 2a,b). Upon activation, B cells from *Cd19Rfx7*^fl/fl^*Myc*^fl/fl^ mice showed lower expression of Myc targets as compared to *Rfx7*^−/−^ B cells and markedly reduced Myc levels, confirming its deletion (Extended Data Fig. 3b). It can also be appreciated that, although the effects of Rfx7-deficiency on Myc targets are consistent with previous data, those on Myc levels are not observed, suggesting that Rfx7 acts on Myc activity rather than Myc levels (Extended Data Fig. 3b). Notably, whereas FO B cells from *Cd19Rfx7*^fl/fl^*Myc*^fl/fl^ mice were almost unresponsive to CpG or anti-IgM stimulation, FO B cells from *Cd19Rfx7*^fl/fl^*Myc*^fl/wt^ mice showed normalized blasting (Fig. 3a), in agreement with the role of Myc in regulating lymphocyte granularity upon activation and substantiating its role in the enhanced activation of Rfx7-deficient B cells^34^.

Given the relevance of Myc and the increased *RFX7* transcript levels in GC B cells^19–21^ (Fig. 2a), we next assessed the role of Rfx7 following immunization of *Cd19Rfx7*^fl/fl^ and *Cd19Rfx7*^wt/wt^ mice. Lack of Rfx7 led to increased frequency and number of GC B cells (Fig. 3b and Extended Data Fig. 3c). Immunofluorescence staining with the GL-7 antibody revealed a trend towards larger GC area in immunized *Cd19Rfx7*^fl/fl^ mice, as compared to *Cd19Rfx7*^wt/wt^ mice (Fig. 3c and Extended Data Fig. 3d). As compared to *Rfx7*^+/+^ GC B cells, their *Rfx7*^−/−^ counterparts expressed more homogenously the proliferative marker Ki67 and presented increased side scatter (SSC), indicative of cell content granularity (Fig. 3b and Extended Data Fig. 3c). Moreover, they did not present significant differences in the relative abundance of dark zone (DZ) and light zone (LZ) GC subsets (Extended Data Fig. 3c). Notably, immunized *Cγ1CreRfx7*^fl/fl^ mice^35^, further crossed onto a Cre-reporter (YFP) strain, showed an increased proportion of YFP-positivity among total and GC B cells (with a tendency to have a DZ phenotype) as compared to control mice, indicating that the effects of Rfx7 on activated and GC B cells were recapitulated when its deletion was coupled to antigenic stimulation (Fig. 3d). Immunized *Cd19Rfx7*^fl/fl^ mice also showed increased frequency and number of PBs, but not of plasma cells (PCs; Fig. 3b). As with GC B cells, *Rfx7*^−/−^ PBs and PCs presented increased SSC (Fig. 3b). *Cd19Rfx7*^fl/fl^*Myc*^fl/wt^ mice showed normalized GC B cells, PB percentages and SSC in these subsets (Fig. 3b), highlighting the role of Rfx7 in restricting Myc activity.

### Rfx7 delays development of p53-loss-driven lymphoma

**Fig. 4:**
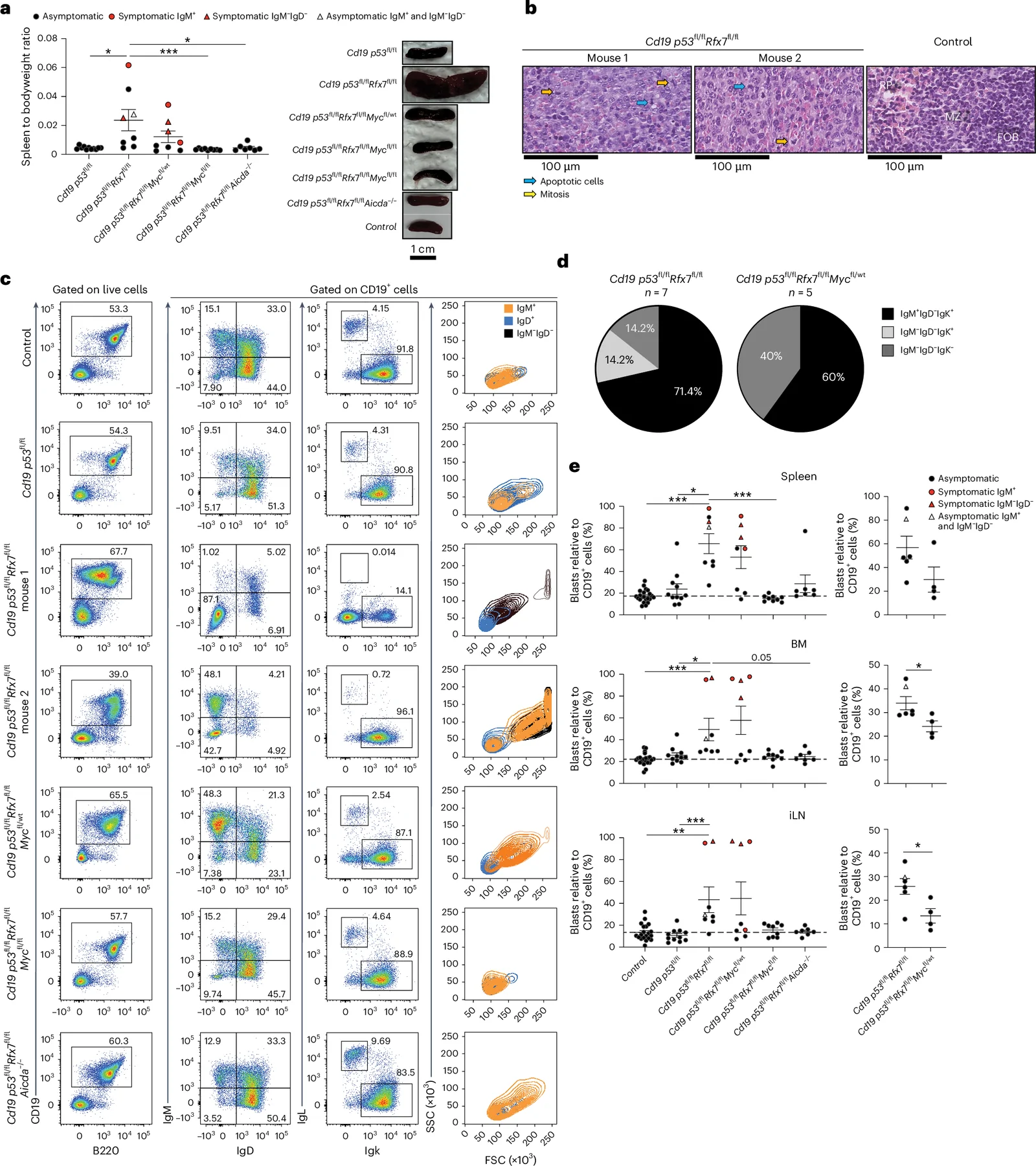
Rfx7 deficiency accelerates lymphoma development in a p53 knockout model. **a**–**e**, Cre-negative control (*n* = 22, for iLNs *n* = 20), *Cd19p53*^fl/fl^ (*n* = 11), *Cd19p53*^fl/fl^*Rfx7*^fl/fl^ (*n* = 8), *Cd19p53*^fl/fl^*Rfx7*^fl/fl^*Myc*^fl/wt^ (*n* = 8), *Cd19p53*^fl/fl^*Rfx7*^fl/fl^*Myc*^fl/fl^ (*n* = 9) and *Cd19p53*^fl/fl^*Rfx7*^fl/fl^*Aicda*^−/−^ (*n* = 7) mice were analyzed at 300 days or earlier in case of overt disease symptoms. Mice asymptomatic until the endpoint are identified by black or white symbols (**a** and **e**); mice euthanized before the endpoint are classified as ‘symptomatic’ and are identified by red symbols (**a** and **e**). Mice with IgM^+^ tumors are identified by circular symbols, those with IgM/IgD^−^ by triangular symbols; the mouse presenting lymphoma cells with two distinct phenotypes is represented by an empty triangular symbol (**a** and **e**). Quantification of spleen weight over bodyweight and representative spleen images (**a**), H&E staining of a control and two diseased *Cd19p53*^fl/fl^*Rfx7*^fl/fl^ mice (**b**), representative flow cytometry analysis of the spleens of indicated genotypes (**c**), pie charts illustrating Ig distribution among evaluated lymphomas (**d**), graphs showing the percentage of blastoid cells among B cells in spleen, iLNs or BM of all analyzed mice (**e**, left) or of mice analyzed at the endpoint (**e**, right) are depicted. Results represent mean ± s.e.m. and statistical comparisons used Kruskal–Wallis test comparing *Cd19p53*^fl/fl^*Rfx7*^fl/fl^ to the other groups; only significant results are shown (**a** and **e**); *P ≤ 0.05, **P ≤ 0.01, ***P ≤ 0.001. Dashed lines in the graph represent the mean of control group (**e**). FOB, FO B cell zone; MZ, MZ zone; RP, red pulp zone. Source data

**Extended Data Fig. 4:**
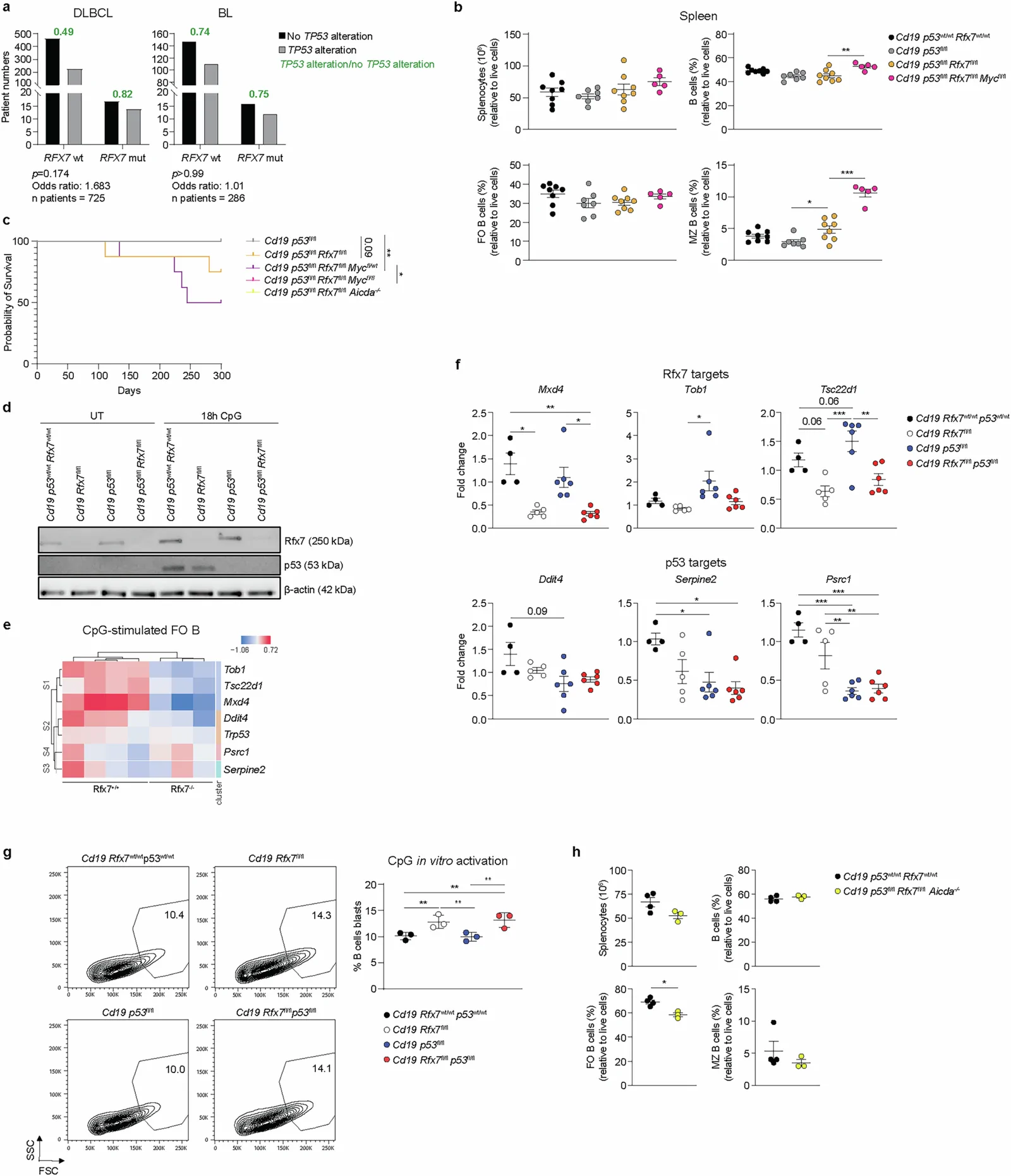
B cell compartment in mice lacking p53, Rfx7, and Myc. **a**) The number and ratio of BL or DLBCL patients^10,11,16,52–54^ with and without *TP53* alterations are shown according to *RFX7* mutation status (related to Supplementary Table 3). Fisher’s exact test (two-sided); odds ratio (OR, 95% CI). **b**) The B cell compartment of 6–16 weeks old *Cd19p53*^wt/wt^*Rfx7*^wt/wt^, *Cd19p53*^fl/fl^, *Cd19p53*^fl/fl^*Rfx7*^fl/fl^, and *Cd19p53*^fl/fl^*Rfx7*^fl/fl^*Myc*^fl/fl^ mice was analyzed at steady state. Graphs illustrate spleen cellularity, B cell (CD19^+^B220^+^), follicular B cell (CD21^−^CD23^+^), and marginal zone B cell (CD21^+^CD23^−^) percentages. Results represent mean±s.e.m. of n = 8 *Cd19p53*^wt/wt^*Rfx7*^wt/wt^, n = 7 *Cd19p53*^fl/fl^, n = 8 *Cd19p53*^fl/fl^*Rfx7*^fl/fl^, and n = 5 *Cd19p53*^fl/fl^*Rfx7*^fl/fl^*Myc*^fl/fl^ mice and are a pool of two independent experiments. Statistical comparisons were performed using one-way ANOVA corrected for multiple testing using the Dunnett method; only significant results between *Cd19p53*^fl/fl^*Rfx7*^fl/fl^ mice and the other groups are shown. **c**) Survival curves of n = 11 *Cd19p53*^fl/fl^, n = 8 *Cd19p53*^fl/fl^*Rfx7*^fl/fl^, n = 8 *Cd19p53*^fl/fl^*Rfx7*^fl/fl^*Myc*^fl/wt^, n = 9 *Cd19p53*^fl/fl^*Rfx7*^fl/fl^*Myc*^fl/fl^, and n = 7 *Cd19p53*^fl/fl^*Rfx7*^fl/fl^*Aicda*^*−/−*^ mice. Mice were sacrificed at the onset of symptoms or at the end of the experiment at 300 days. Statistical comparisons between the experimental groups were performed using Mantel Cox test; only significant comparisons are shown. **d**) Immunoblot analysis showing the expression of Rfx7 and p53 in lysates of untreated or CpG-stimulated (18 h) B cells enriched from spleens of *Cd19p53*^wt/wt^*Rfx7*^wt/wt^, *Cd19Rfx7*^fl/fl^, *Cd19p53*^fl/fl^, and *Cd19p53*^fl/fl^*Rfx7*^fl/fl^ mice. β-actin was used as loading control and data are representative of two independent experiments. **e**) Heatmap of a selection of genes (*Tob1, Tsc22d1*, *Mxd4*, *Ddit4*, *Trp53, Psrc1*, and *Serpine2*) from RNA-seq analysis presented in Fig. 2d is shown. **f**) Sorted splenic FO B cells (CD19^+^, B220^+^ and CD23^+^) of 6 to 14-week-old mice of indicated genotypes were stimulated with CpG (18 h). Graphs show mRNA levels of *Mxd4*, *Tob1*, *Tsc22d1*, *Ddit4*, *Serpine2*, and *Psrc1* assessed by RT-qPCR (values normalized to *Hprt* and *18* *s*; results are pool of two experiments, one control from each experiment was set to 1). Results represent mean ± s.e.m. of n = 4 *Cd19Rfx7*^wt/wt^, n = 5 *Cd19Rfx7*^fl/fl^, n = 6 *Cd19p53*^fl/fl^, and n = 6 *Cd19p53*^fl/fl^*Rfx7*^fl/fl^ mice. Statistical comparisons were performed using two-way ANOVA corrected for multiple testing using Tukey method; only significant comparisons are shown. **g**) Graphs show percentage of blasting B cells of indicated genotypes, 18 h post-stimulation with CpG, and the representative flow cytometry plots thereof (results represent mean±s.e.m. of n = 3 mice/group). Statistical comparisons were performed using two-way ANOVA corrected for multiple testing using Tukey method; only significant comparisons are shown. **h**) The B cell compartment of 6–14 weeks old *Cd19p53*^wt/wt^*Rfx7*^wt/wt^ and *Cd19p53*^fl/fl^*Rfx7*^fl/fl^*Aicda*^*−/−*^ mice was analyzed at steady state. Graphs illustrate spleen cellularity, B cell (CD19^+^B220^+^), follicular B cell (CD21^−^CD23^+^), and marginal zone B cell (CD21^+^CD23^−^) percentages. Results represent mean±s.e.m. of n = 4 *Cd19p53*^wt/wt^*Rfx7*^wt/wt^ and n = 3 *Cd19p53*^fl/fl^*Rfx7*^fl/fl^*Aicda*^*−/−*^ mice. Statistical comparisons were performed using unpaired student’s t-test, two-tailed. Source data

As *TP53* (the human gene encoding p53) aberrations are found in 30–70% of BL and DLBCL cases presenting *RFX7* mutations^14^ (Supplementary Table 3 and co-occurrence analyses in Extended Data Fig. 4a) and a TP53–RFX7 signaling axis was reported in other cancer types^5^, we evaluated the contribution of Rfx7-loss and Myc (haplo-)insufficiency in a lymphoma model with B cell-specific deletion of p53 (encoded by the *Trp53* gene, referred to here as *p53*), which causes IgM^+^ MZ expansion progressing towards DLBCL^36,37^. At a young age, *Cd19p53*^fl/fl^, *Cd19p53*^fl/fl^*Rfx7*^fl/fl^, *Cd19p53*^fl/fl^*Rfx7*^fl/fl^*Myc*^fl/fl^ and control mice did not present substantial differences in spleen size and B cell percentage except for MZ B cells, which were increased mildly in *Cd19p53*^fl/fl^*Rfx7*^fl/fl^ as compared to *Cd19p53*^fl/fl^ mice and further increased by Myc ablation (Extended Data Fig. 4b). We thus monitored these mice over time for tumor development. Mice that were asymptomatic until the endpoint (300 days) are displayed by black or white symbols (Fig. 4a,e); mice that were euthanized at the appearance of disease symptoms before the endpoint are instead represented by red symbols (Fig. 4a,e). As compared to *Cd19p53*^fl/fl^ mice, *Cd19p53*^fl/fl^*Rfx7*^fl/fl^ mice exhibited enlarged spleens and two out of eight cases of symptomatic disease (Fig. 4a and Extended Data Fig. 4c). Histologic examination of spleens from asymptomatic *Cd19p53*^fl/fl^*Rfx7*^fl/fl^ mice with moderately enlarged spleens already revealed loss of architecture and medium-to-large cell populations with vesicular and enlarged nuclei, congruent with a high-grade lymphoma (Fig. 4b).

Based on these, we probed the relationship between p53 and Rfx7. Both proteins were expressed by in vitro activated B cells without exhibiting obvious interdependence, in line with *p53* not being altered in our RNA-seq data and *Rfx7* not being reported as p53 target in B cells^38^ (Extended Data Fig. 4d,e). We next assessed the mRNA levels of p53 and Rfx7 target genes (based on published^3,5,38^ and our own data; Extended Data Fig. 4e). Whereas the Rfx7 targets—in particular *Mxd4* and *Tsc22d1*, reported to have tumor suppressor functions^39–41^—were decreased in Rfx7-deficient B cells but not in p53^−/−^ B cells, the p53 targets *Ddit4, Serpine2* and *Psrc1* were variably modulated by Rfx7 (*Ddit4* is also a direct Rfx7 target; Extended Data Fig. 4f). Moreover, following CpG stimulation, the enhanced blasting caused by Rfx7 deletion was similar on a p53^−/−^ and p53^+/+^ background (Extended Data Fig. 4g). These findings suggest that RFX7 might contribute to the p53 signature, while regulating additional p53-independent pathways.

### Myc and AID contribute to *p53*^−/−^*Rfx7*^−/−^ B cell lymphoma

Although *Cd19p53*^fl/fl^*Rfx7*^fl/fl^*Myc*^fl/wt^ mice also exhibited enlarged spleens and symptomatic disease, *Cd19p53*^fl/fl^*Rfx7*^fl/fl^*Myc*^fl/fl^ mice were protected from lymphoma (Fig. 4a and Extended Data Fig. 4c). Flow cytometry analyses revealed the presence of B cell populations with variably altered CD19/B220 staining in *Cd19p53*^fl/fl^*Rfx7*^fl/fl^ and *Cd19p53*^fl/fl^*Rfx7*^fl/fl^*Myc*^fl/wt^ mice (Fig. 4c). Such populations exhibited marked blastoid morphology and were mostly positive for IgM (Fig. 4c–e). In 4 out of 12 cases, lymphoma cells were IgM^−^IgD^−^, with one positive for kappa light chain, compatibly with a switched PB phenotype (Fig. 4c,d). Only one *Cd19p53*^fl/fl^*Rfx7*^fl/fl^ mouse exhibited a mix of two distinct Ig expression patterns, suggesting the existence of at least two clones (Fig. 4c; empty triangular symbols in Fig. 4a,e). *Cd19p53*^fl/fl^ mice showed no or mild alterations of CD19/B220 staining, with few mice presenting with IgM^+^ blastoid cells in the spleen but negligibly in iLN and bone marrow (BM) (Fig. 4c,e). *Cd19p53*^fl/fl^*Rfx7*^fl/fl^ and *Cd19p53*^fl/fl^*Rfx7*^fl/fl^*Myc*^fl/wt^ mice exhibited instead spreading of blasts to LNs and BM, a phenomenon associated with symptomatic disease (Fig. 4e, left). When focusing on mice euthanized at endpoint, *Myc* heterozygosity trended towards or significantly reduced the frequency of blastoid B cells in spleen, iLN and BM (Fig. 4e, right). These data indicate that loss of Rfx7 promotes lymphomagenesis whereas Myc deletion blocks it and Myc heterozygosity displays a protective effect on nonsymptomatic disease.

Myc deregulation occurs commonly due to chromosome translocations caused by the aberrant activity of AID (encoded by the *Aicda* gene)^22,42–44^. We therefore asked whether AID contributes to lymphomagenesis of B cells on a *p53*^−/*−*^*Rfx7*^−/−^ background. At a young age, *Cd19p53*^fl/fl^*Rfx7*^fl/fl^*Aicda*^−/−^ and control mice did not present substantial differences in spleen size and B cell percentage except for FO B cells, which were decreased mildly (Extended Data Fig. 4h). Notably, *Cd19p53*^fl/fl^*Rfx7*^fl/fl^*Aicda*^−/−^ mice were asymptomatic until the endpoint; six out of seven mice presented negligible alterations of the B cell compartment, whereas one mouse exhibited a mildly enlarged spleen with IgM^+^ blastoid cells not spreading to iLNs and BM (Fig. 4a,c,e and Extended Data Fig. 4c). This indicates that AID is not absolutely required for lymphomagenesis driven by p53 and Rfx7 loss. Yet, protection from advanced disease provided by its deficiency reveals the importance of B cell activation and AID activity in the trajectory promoted by Rfx7 loss.

### *p53*^−/−^*Rfx7*^−/−^ lymphoma cells exhibit largely PB and PC features

**Fig. 5:**
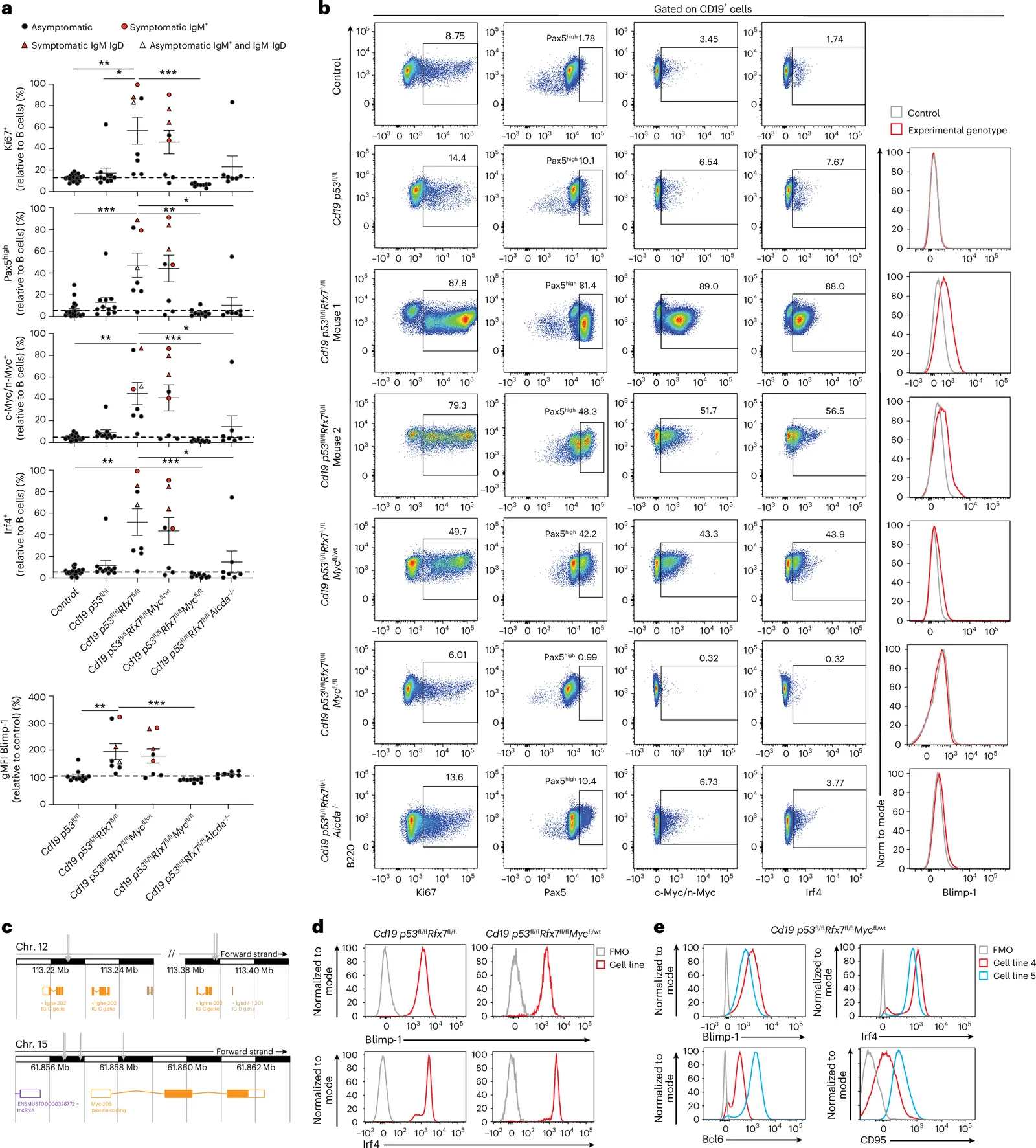
Rfx7 deficiency promotes B cell lymphoma characterized by deregulated Myc expression. **a**,**b**, Mice and symbols are as described in Fig. 5a,e. Graphs show percentage positive relative to B cells for c-Myc/n-Myc, Pax5, Ki67, Irf-4 and Blimp-1 geometric mean fluorescence intensity in B cells of experimental mice relative to controls analyzed on the same day in the spleen (**a**). Representative flow cytometry analyses of the spleens of the indicated genotypes are shown; for Blimp-1, red lines in histograms represent experimental genotype; gray lines represent respective controls (**b**). Results represent mean ± s.e.m. of *n* = 22 control, *n* = 11 *Cd19p53*^fl/fl^, *n* = 8 *Cd19p53*^fl/fl^*Rfx7*^fl/fl^, *n* = 8 *Cd19p53*^fl/fl^*Rfx7*^fl/fl^*Myc*^fl/wt^, *n* = 9 *Cd19p53*^fl/fl^*Rfx7*^fl/fl^*Myc*^fl/fl^, *n* = 7 *Cd19p53*^fl/fl^*Rfx7*^fl/fl^*Aicda*^−/−^ mice/group. Statistical comparisons used Kruskal–Wallis test comparing *Cd19p53*^fl/fl^*Rfx7*^fl/fl^ to the other groups; only significant comparisons are shown (**a**). Dashed lines in the graph represent the mean of the control group (**a**); *P ≤ 0.05, **P ≤ 0.01, ***P ≤ 0.001. **c**–**e**, Cell lines generated from *Cd19p53*^fl/fl^*Rfx7*^fl/fl^ and *Cd19p53*^fl/fl^*Rfx7*^fl/fl^*Myc*^fl/wt^ mice were characterized. The location of the identified Myc/IgH breakpoints is indicated within the Myc and IgH loci with arrows (**c**). Flow cytometry plots illustrate Blimp-1 and Irf-4 expression in representative cell lines of the indicated genotypes (**d**). Histogram overlays show expression levels of Blimp-1, Irf-4, Bcl6 and CD95 in a cell line harboring GC features (blue) and a cell line with a more plasmablastic phenotype (red) (**e**). FMO, fluorescence minus one (gray) (**d** and **e**). Source data

**Extended Data Fig. 5:**
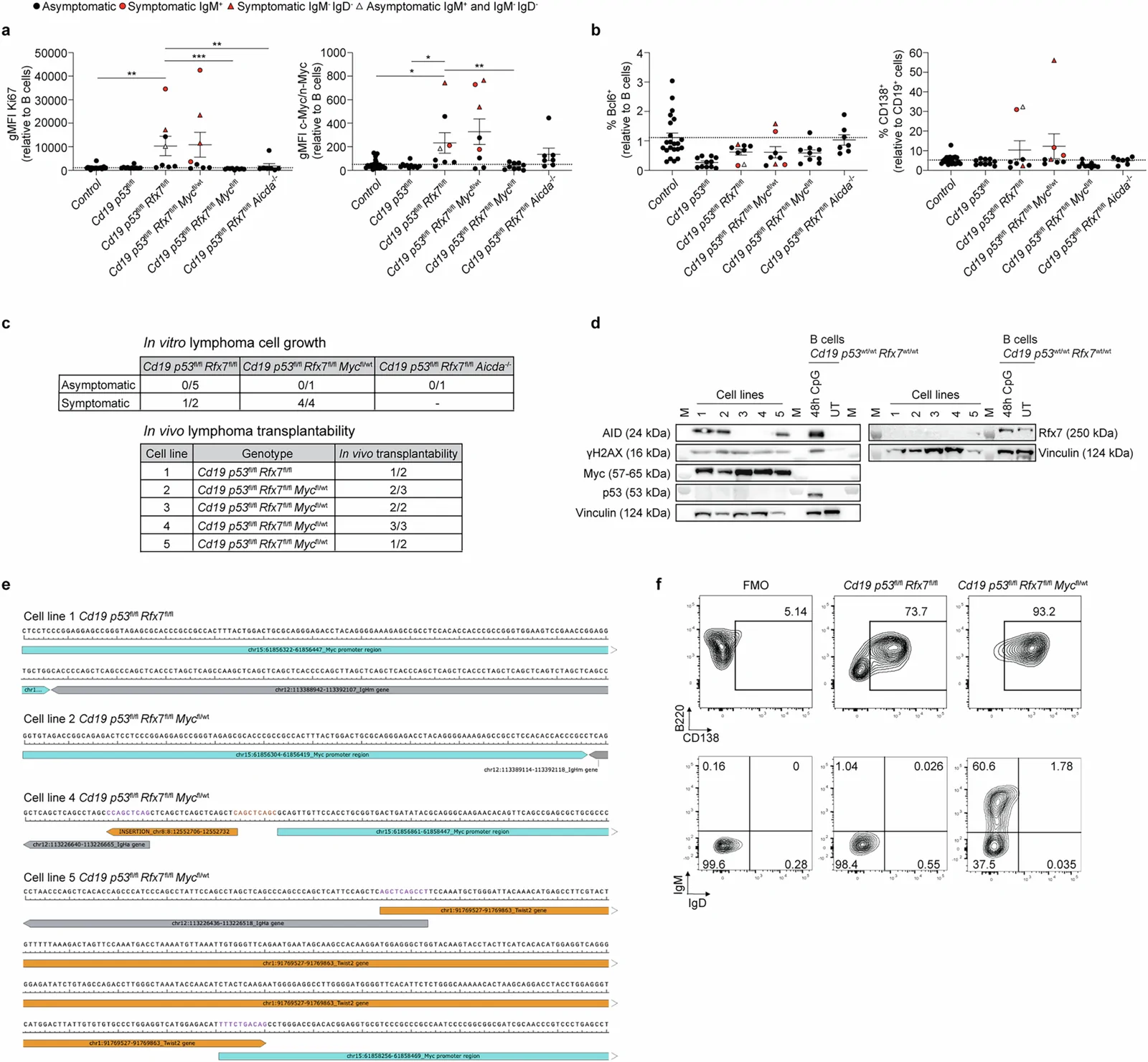
Phenotypic characterization of lymphoma cells and cell lines. **a** and **b**) Mice and symbols are the same as described for Fig. 4b and f. Graphs show mean fluorescence intensity (gMFI) relative to B cells for c-Myc/n-Myc and Ki67 (**a**). Graph illustrates percentage Bcl6- and CD138-positive cells among splenic B cells (**b**). Dotted lines in the graph represent the mean of control group. Results represent mean±s.e.m. of n = 22 control, n = 11 *Cd19p53*^fl/fl^, n = 8 *Cd19p53*^fl/fl^*Rfx7*^fl/fl^, n = 8 *Cd19p53*^fl/fl^*Rfx7*^fl/fl^*Myc*^fl/wt^, n = 9 *Cd19p53*^fl/fl^*Rfx7*^fl/fl^*Myc*^fl/fl^, n = 7 *Cd19p53*^fl/fl^*Rfx7*^fl/fl^*Aicda*^*−/−*^ mice/group; statistical comparisons were performed with Kruskal-Wallis test comparing *Cd19p53*^fl/fl^*Rfx7*^fl/fl^ to the other groups and only significant comparisons are shown (**a** and **b**). **c**) Splenic cells from diseased *Cd19p53*^fl/fl^*Rfx7*^fl/fl^, *Cd19p53*^fl/fl^*Rfx7*^fl/fl^*Myc*^fl/wt^, and *Cd19p53*^fl/fl^*Rfx7*^fl/fl^*Aicda*^*−/−*^ mice were cultured *in vitro* as explained in the Materials and Methods section. The upper table illustrates the number of cultures giving rise to ‘cell lines’ able to expand for over two weeks. The lower table shows the *in vivo* transplantability of the five lymphoma cell lines in immunocompetent mice. **d**) Immunoblot analysis shows expression of AID, γH2AX, Myc, p53, and Rfx7 in cell lysates from the indicated cells lines, as well as enriched splenic B cells from *Cd19p53*^wt/wt^*Rfx7*^wt/wt^ mice left untreated (UT) or stimulated with CpG for 48 h. Vinculin is shown as loading control. **e**) Nucleotides surrounding each breakpoint and homologous germline regions are indicated. Microhomology regions are highlighted in purple and duplications thereof in brown. **f**) Flow cytometry plots show B2220, CD138, IgM, and IgD expression in established cell lines of the indicated genotypes. Source data

Molecular phenotyping revealed that lymphoma cells on a *p53*^−/−^*Rfx7*^−/−^ background—especially from symptomatic mice—exhibited high levels of Ki67, paired box 5 (Pax5)^45^ and Myc expression, indicating a clear deregulation of Myc even in diseased *Cd19p53*^fl/fl^*Rfx7*^fl/fl^*Myc*^fl/wt^ mice (Fig. 5a,b and Extended Data Fig. 5a). As expected, Myc was negligible in *Cd19p53*^fl/fl^*Rfx7*^fl/fl^*Myc*^fl/fl^ mice, whereas it was expressed at levels comparable to *p53*^−/−^ B cells in most *Cd19p53*^fl/fl^*Rfx7*^fl/fl^*Aicda*^−/−^ mice (Fig. 5a,b and Extended Data Fig. 5a). This suggests that AID promotes deregulation of pro-tumorigenic genes in *p53*^−/−^*Rfx7*^−/−^ B cells.

We next assessed the capacity of lymphoma cells on a *p53*^*−/−*^*Rfx7*^*−/−*^ background from asymptomatic and symptomatic diseased mice to proliferate in vitro in the absence of specific growth factors. Even though *Cd19p53*^fl/fl^*Rfx7*^fl/fl^ and *Cd19p53*^fl/fl^*Rfx7*^fl/fl^*Myc*^fl/wt^ mice presented a limited number of symptomatic lymphomas, most of them grew in vitro, something that was not observed for nonsymptomatic disease (Extended Data Fig. 5c). The established ‘cell lines,’ which were transplantable in immunocompetent syngeneic mice, lacked Rfx7 and p53 expression, but exhibited high Myc and variable AID levels (Extended Data Fig. 5c,d). In line with the presence of genotoxic stress, we detected γH2AX positivity and polymerase chain reaction (PCR) products indicative of chromosome translocations between the IgH and Myc loci in four out of five cell lines (Fig. 5c and Extended Data Fig. 5d,e), supporting the involvement of AID in the progression of the accelerated lymphomas on the *p53*^−/−^*Rfx7*^−/−^ background.

These lines were mono-clonal based on CDR3-sequencing and largely retained the original phenotype, with IgM^+^ tumors giving rise to both IgM^+^ and IgM^−^ fractions (Extended Data Fig. 5f and Supplementary Table 1b). Whereas ex vivo only few tumors presented CD138 positivity, the cell lines were all positive for this dynamically expressed PB/PC marker^46^ (Extended Data Fig. 5b,f). Notably, B cells from *Cd19p53*^fl/fl^*Rfx7*^fl/fl^ and *Cd19p53*^fl/fl^*Rfx7*^fl/fl^*Myc*^fl/wt^ were largely negative for Bcl6 and positive for Irf-4 and—particularly in the symptomatic cases—for B lymphocyte-induced maturation protein-1 (Blimp-1) (Fig. 5a,b and Extended Data Fig. 5b). Irf-4 and Blimp-1 were expressed also by the derived cell lines, with one line (line 5) presenting lower Irf-4 and Blimp-1 levels and positive for Bcl6 and CD95, compatible with a GC origin (Fig. 5d,e). These results underscore how lymphoma cells on a *p53*^−/−^*Rfx7*^−/−^ background combine aggressive proliferative features with markers associated to GC or PB/PC differentiation^47,48^.

### RFX7 nonsense mutations cause loss of function

**Fig. 6:**
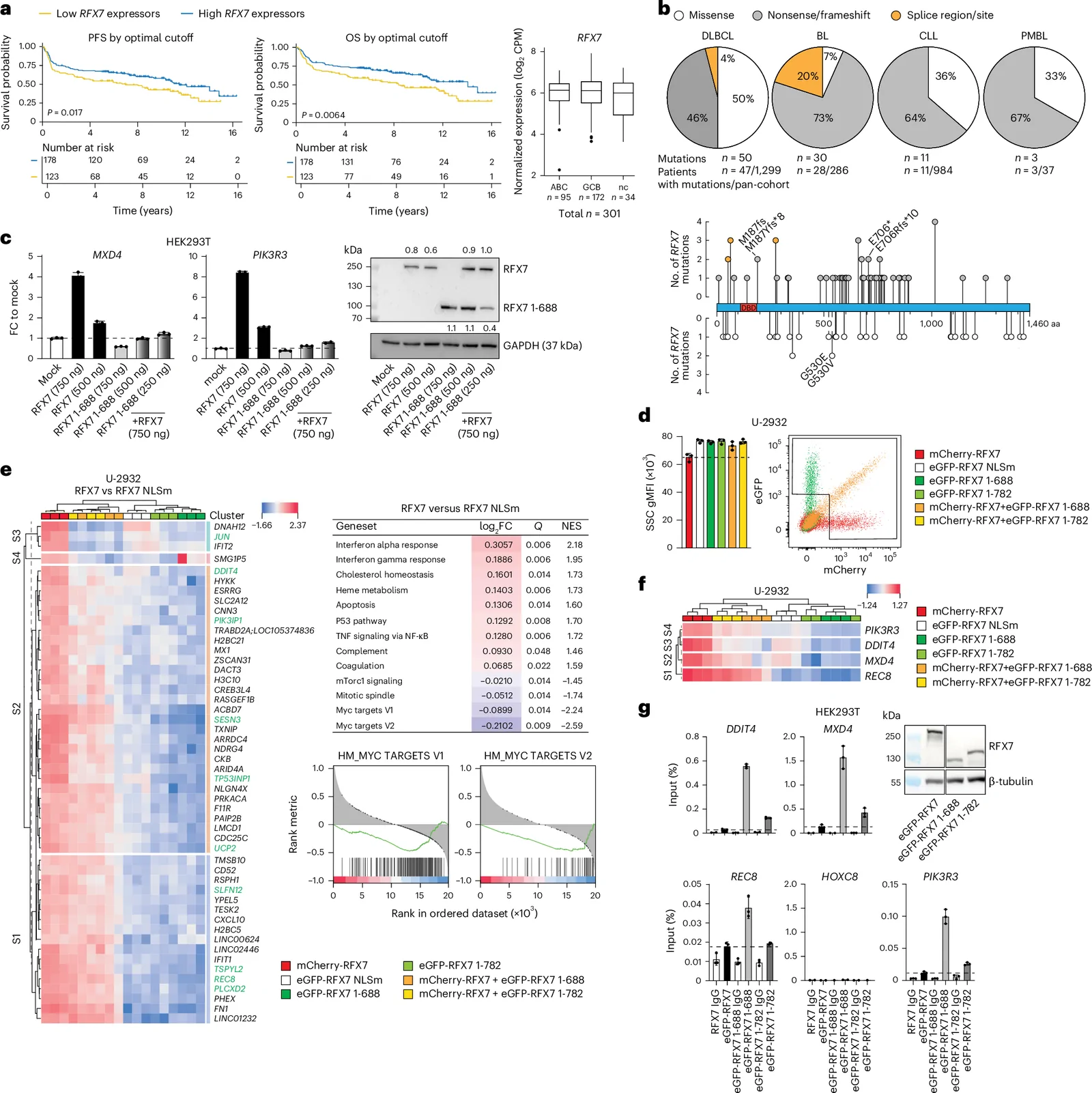
RFX7 mutants found in lymphoid cancers are dysfunctional. **a**, To evaluate associations with PFS and OS, normalized *RFX7* mRNA values from DLBCLs^50^ were dichotomized into high and low-expression groups with an optimal cutoff and used for Kaplan–Meier analysis and Cox modeling; statistical analysis used the two-tailed log-rank test. Right: graph depicting *RFX7* mRNA values in activated B cell-like (ABC), GC B cell-like (GCB) or unclassified lymphomas (nc). Data are presented as box-and-whisker plots; center line: median; box limits: interquartile range. **b**, Pie charts illustrate the type of identified *RFX7* mutations, the number of patients with such mutations over the total number of patients across the indicated lymphoid malignancies and a lollipop plot of mutations across RFX7 (related to Supplementary Table 3). **c**, HEK293T cells were cotransfected with plasmids encoding eGFP and empty plasmid (mock), Myc-tagged RFX7 and RFX7 1–688 (two doses) as indicated. eGFP-positive cells were sorted and *MXD4* and *PIK3R3* mRNA abundance was analyzed by RT–qPCR (data are plotted as FC relative to mock-transfected cells, normalized to *POLR2A*). RFX7 expression was analyzed by immunoblot using an αMyc antibody; intensity of RFX7 bands relative to GAPDH is reported. **d**–**f**, U-2932 cells were transfected with plasmids encoding N-terminally eGFP- or mCherry-tagged WT, NLSm, 1–688, 1–782 RFX7 and combinations thereof, as indicated. Cells positive for mCherry and/or eGFP were analyzed for SSC by flow cytometry (**d**) and by RNA-seq (**e**). Hierarchical clustering of the top 60 significantly differentially expressed genes (DEG); statistical analysis compared RFX7 versus NLSm RFX7 using DESeq2 with the Wald test (FDR = 0.05 and log_2_FC = 1; green, reported RFX7 targets^3,5,30^). Hallmark genesets (MSigDB; *Q* < 0.05 using fGSEA), and selected GSEA plots are shown (**e**). Hierarchical clustering of the four indicated genes is illustrated (**f**). **g**, HEK293T cells were transfected with eGFP-tagged WT, 1–688 and 1–782 RFX7. Chromatin extracted from eGFP-positive cells was immunoprecipitated with αGFP or isotype control antibody followed by qPCR for the indicated genes’ promoters. RFX7 expression was analyzed by immunoblot using an αRFX7 antibody. Results show *n* = 1–3 technical replicates (**c**–**g**) and are represented as mean ± s.d. (**c**, **d** and **g**). Source data

**Extended Data Fig. 6:**
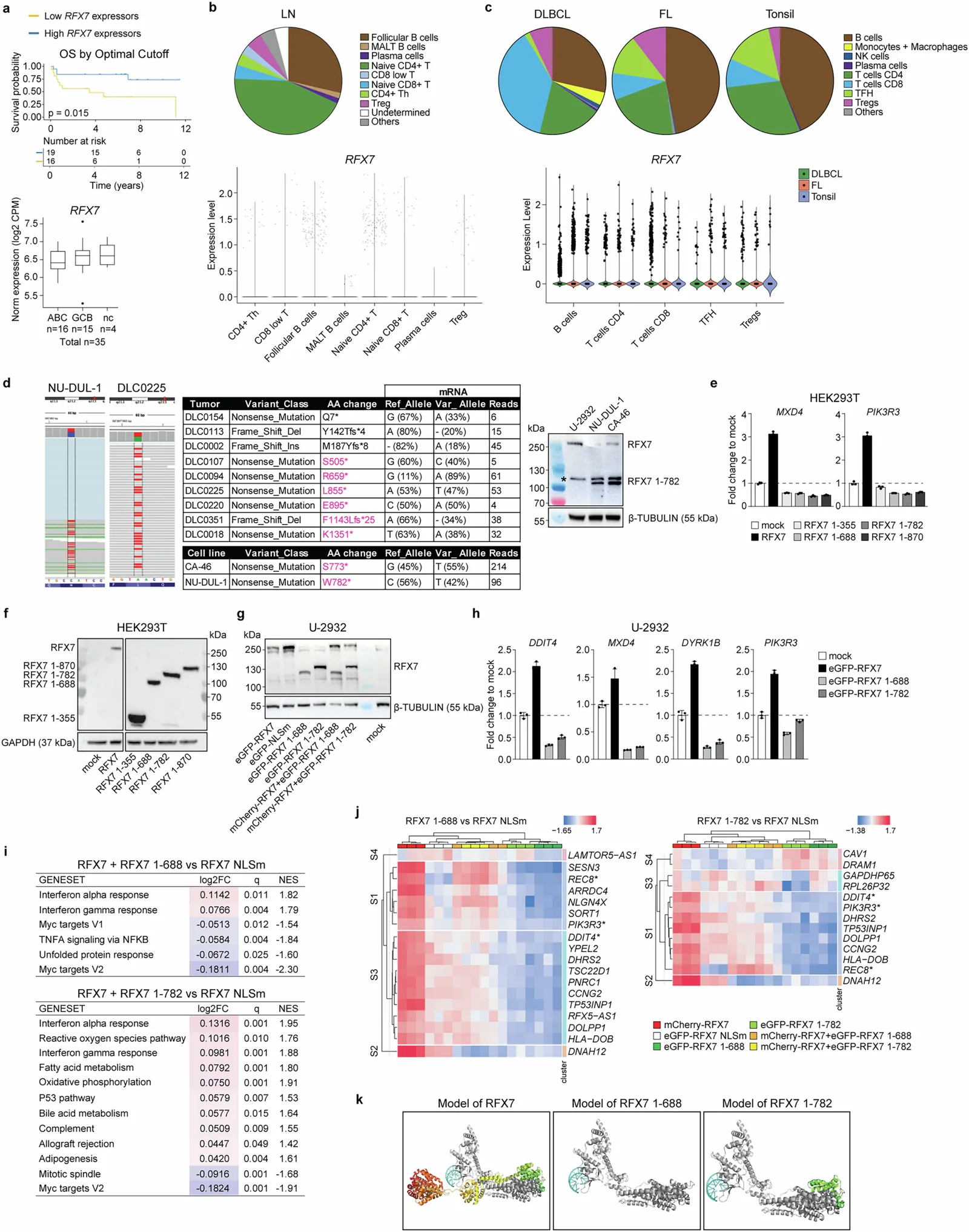
Expression and effects of RFX7 mutants. **a**) To evaluate associations with Overall Survival (OS), normalized *RFX7* mRNA values from DLBCLs^49^ were dichotomized into high and low expression groups with an optimal cutoff and used for Kaplan–Meier analysis and Cox modelling; statistical analysis was performed by using two-tailed Log-rank test. The graph on the right depicts *RFX7* mRNA values in ABC, GCB, or unclassified lymphomas and presents the data as a box-and-whisker plot; the center line defines the median and the box limits represents interquartile range (IQRs). **b**, **c**) Publicly available single-cell RNAseq data from normal LNs (n = 10, GSE131907^83^) (**b**), or tonsil (n = 1), follicular lymphoma (FL, n = 3), and DLBCL biopsies (n = 4, GSE182434^51^) (**c**) were analyzed for cell subsets (representing >1% of total cells (**b**); upper panels) and for *RFX7* expression levels (log-normalized expression, lower panels) in the major immune subsets, as defined in the original publication. **d**) Integrative Genomics Viewer (IGV) analysis^84^ of the mRNA around the mutation, for the NU-DUL-1 lymphoma line and the patient sample DLC0225; red, variant allele; green/blue, reference allele (left panel). Table showing the RFX7 aminoacidic mutation and percentage of reference and variant allele at mRNA level, analysed with IGV, from patient’s samples (from BC Cancer dataset EGAD00001003783) and lymphoma cell lines (NU-DUL-1: GEO GSM7789550; CA-46: GEO GSM8927146; middle panel; patients samples were annotated with GRCh37, CA-46 and NU-DUL-1 with GRCh38; residues codified by the last exon are highlighted in pink). RFX7 protein level was analyzed using an antibody recognizing RFX7 N-terminus in lysates from the indicated cell lines (*= aspecific band). **e** and **f**) HEK293T cells were cotransfected with empty vector (mock), Myc-DDK-tagged full-length human RFX7, or the indicated truncated versions of RFX7 and eGFP-encoding vectors. eGFP-positive cells were sorted, *MXD4* and *PIK3R3* mRNA abundance was analyzed by qRT-PCR (e; data are plotted as fold change relative to mock transfected cells, normalized to *POLR2A;* results show n = 3 technical replicates and are represented as mean ± s.d.) and expression of the transfected proteins was analyzed using immunoblot with anti-Myc-tag antibody (**f**). **g** and **h**) U-2932 cells were transfected with plasmids encoding N-terminally eGFP- or mCherry-tagged full-length, NLSm, 1-688, 1-782 RFX7, and combinations thereof, as indicated. Expression was analyzed using an antibody recognizing RFX7 N-terminus (**g**). eGFP-positive cells were sorted and tested for the abundance of *DDIT4*, *MXD4, DYRK1B*, and *PIK3R3* mRNA by qRT-PCR (data are plotted as fold change relative to mock transfected cells, normalized to *POLR2A*); results are represented as mean ± s.d. of n = 3 technical replicates (**h**). **i** and **j**) U-2932 cells were transfected with plasmids encoding N-terminally eGFP- or mCherry-tagged full-length, NLSm, 1-688, 1-782 RFX7, and combinations thereof, as indicated. Cells positive for mCherry and/or eGFP were sorted and analyzed by RNA-sequencing (RNAseq). Hallmark gene sets altered in the indicated comparisons (MSigDB; q < 0.05 using fGSEA) are shown (**i**). Hierarchical clustering of significantly differentially expressed genes in the indicated comparisons is illustrated (**j**). **k**) Homology models of RFX7 isoforms; full length (left), 1-688 (center) and 1-782 (right), in complex with dsDNA (teal). Residues 689-782 (in RFX7 1-782) and 689–1460 (in RFX7 full length), located in the proximity of the dsDNA, are in color. Source data

Finally, we aimed to better understand the impact of RFX7 alterations in human lymphoid malignancies. We analyzed overall survival (OS) and progression free survival (PFS) relative to *RFX7* levels (which are not altered in different DLBCL subtypes, Fig. 6a) in DLBCL. Notably, high *RFX7* expression associated with better prognosis in two DLBCL cohorts of 301 and 35 patients^49,50^ (Fig. 6a and Extended Data Fig. 6a), suggesting that *RFX7* transcript abundance impacts on disease outcomes. Multivariate Cox analysis for OS and PFS demonstrated that the *RFX7* classification is independent of the cell of origin (hazard ratio (HR) = 1.55 (low-expression group) and *P* = 0.01 for OS, HR = 1.46 and *P* = 0.02 for PFS) for the larger cohort (HR = 3.07 and *P* = 0.06 for OS in the smaller cohort). Although the analyzed biopsies are not restricted to B cells, FO B cells, together with T cells, represent major LN populations and express high levels of *RFX7* transcripts (Extended Data Fig. 6b). Moreover, reanalysis of single-cell RNA-seq data^51^ shows that *RFX7* transcript levels are stable in T and B cells from tonsil, follicular lymphoma—an indolent lymphoma which can transform into DLBCL—and DLBCL, with the exception of DLBCL B cells, which express reduced levels (Extended Data Fig. 6c). These data, which are in line with previous observations^6^, hint to a significant impact of DLBCL cells on *RFX7* mRNA levels in the biopsies used to predict disease outcomes (Fig. 6a and Extended Data Fig. 6a).

We next screened the mutational landscape of *RFX7* in B cell malignancies, by reviewing studies on BL^16,52^, DLBCL^11,12,50,53,54^, CLL^17^ and a small cohort of primary mediastinal large B cell lymphoma^55^, in which we found three *RFX7* mutations corresponding to 8% of patients (Supplementary Table 3). An array of nonsense mutations and frameshifts—distributed over the central portion of the protein, a region encoded largely by the last exon—represented a substantial fraction of *RFX7* alterations (Fig. 6b and Supplementary Table 3). Notably, mRNA arising from the truncations in this exon was detected at approximately the same frequency as the WT allele in DLBCL patients and lymphoma cell lines^16,50^ (Extended Data Fig. 6d). Moreover, we observed a band migrating at the expected molecular size in two lymphoma cell lines, indicating that such premature stop codons are not causing nonsense-mediated decay (Extended Data Fig. 6d).

We thus cloned truncations spanning the central part of RFX7. Overexpression of WT human RFX7 in HEK293T cells increased the transcription of known target genes such as *MXD4* and *PIK3R3*^3^ (Extended Data Fig. 6e). In contrast, the four tested RFX7 mutants failed to do so despite being expressed at high levels (Extended Data Fig. 6e,f). All RFX7 mutants appeared to lower *MXD4* and *PIK3R3* mRNA levels in comparison to mock-transfected cells, suggesting a dominant-negative effect. We therefore selected the 1–688 mutant to test its effects on WT RFX7. Confirming our hypothesis, the 1–688 mutant reduced the capacity of WT RFX7 to transactivate target genes, even when the latter was expressed more abundantly (Fig. 6c).

To approach the question in a more physiological way, we overexpressed WT, 1–688 or 1–782 RFX7 in DLBCL-derived U-2932 cells (Extended Data Fig. 6g). As a negative control, we also included a nuclear localization signal (NLS) mutant (NLSm) version of RFX7^3^. In agreement with the expected effects of RFX7 on cellular metabolism^3^, U-2932 cells overexpressing WT RFX7 exhibited lower SSC compared to the control condition overexpressing NLSm or truncated RFX7 forms (Fig. 6d). Quantitative PCR with reverse transcription (RT–qPCR) and RNA-seq data confirmed the upregulation of known target genes by WT RFX7 (Fig. 6e and Extended Data Fig. 6h). In line with primary B cell results, GSEA corroborated the downregulation of MYC-dependent pathways, whereas we observed the ‘P53 pathway’ among the upregulated signatures^4,5^ (Figs. 2c,d and 6e and Supplementary Table 4). Coexpression of the truncated RFX7 forms (co-expressed at levels comparable to WT RFX7; Extended Data Fig. 6g) counteracted the effects of WT RFX7 on SSC, on MYC targets’ signatures and on the transactivation of a large fraction of target genes, including *DDIT4*, *MXD4*, *PIK3R3* and *REC8* to a smaller extent^3,5^ (Fig. 6d–f and Extended Data Fig. 6i). The truncated RFX7 forms either did not induce target genes or reduced their transcripts below the level of RFX7 NLSm (Fig. 6e); only a negligible number of genes hinted to potential neomorphic features, which were largely lost in the context of coexpression with WT RFX7 (Extended Data Fig. 6j).

In silico analyses predicted that the 1–688 and 1–782 truncations lack protein domains positioning in proximity of the DNA, indicating that the C-terminal part of the protein might regulate chromatin interaction at target gene promoters (Extended Data Fig. 6k). We thus went back to HEK293T cells to test the ability of RFX7 1–688 and 1–782 to bind to target gene promoters^3,5^ by chromatin immunoprecipitation (ChIP) assay. The mutants not only retained the capacity to occupy the promoter of *DDIT4*, *MXD4* and *PIK3R3* but also exhibited an increased ability to do so as compared to WT RFX7 (Fig. 6g). In agreement with a lesser effect of the truncations on *REC8* mRNA abundance, promoter occupancy was similar to the one of WT RFX7, suggesting that *REC8* transactivation has different requirements compared to that of other targets (Fig. 6f,g). Taken together, these results indicate that *RFX7* mutations and transcript abundance can impact on the overall activity of RFX7 and on disease outcomes.

## Discussion

Rfx7 is a poorly studied transcription factor, whose function in the context of healthy and pathologic B cells remains unexplored. Here we uncovered how loss of Rfx7 not only alters normal B cell activation but also represents an advantage for lymphomagenesis.

*RFX7* mutations—mainly nonsense or frameshift mutations—have been identified in various B cell malignancies^6,12–17,50,53,55^. Here we assessed the effects of selected truncations and found them to substantially impair RFX7 transcriptional activity. Moreover, RFX7 truncations showed increased capacity to bind target genes’ promoter antagonizing the functionality of WT RFX7, hinting to the relevance of heterozygous mutations. In silico analyses corroborated an impact of truncated RFX7 forms on DNA binding, urging further investigations to understand RFX7 structure and interactors in lymphoma cells. Moreover, the correlation between *RFX7* transcript levels and DLBCL prognosis highlights RFX7 relevance even in the absence of mutations.

We observed that absence of Rfx7 in the hematopoietic system is per se not sufficient to promote malignant transformation over a substantial timespan. However, when combined with constitutive Bcl6 over-expression or loss of p53 in B cells, Rfx7 deficiency significantly accelerates lymphoma progression. This shows that the tumor suppressor role of Rfx7 is maintained in the absence of p53, extending its relevance to B cell cancers with p53 loss^4,14^. Indeed, our findings suggest that Rfx7 contributes partially to the p53 signature, in line with data from solid cancer cell lines^4,5,56^, while also regulating p53-independent effects.

At the cellular level, Rfx7 limits recently activated GC and PB B cell subsets. RNA-seq, in vitro and in vivo analyses of Rfx7-deficient B cells and RFX7-overexpressing DLBCL cells revealed that RFX7 restricts the expression of genes associated with MYC, proliferation and cancer progression. Notably, Myc haploinsufficiency corrected the exuberant response by *Rfx7*^−/−^ B cells in vitro and in vivo, supporting the role of Myc in their alterations. Although the multiple targets of RFX7 make it difficult to pinpoint a single candidate for limiting MYC activity, MAX dimerization protein 4 (MXD4) certainly stands out^57,58^. Deregulation of feedback loops such as the one involving MXD4 would help explain how RFX7-deficiency is advantageous in Myc-driven mouse models and even in BL^6,13,14,16,59^.

Considering the fairly normal B cell compartment observed in Myc-deficient mice, the fact that deficiency for this transcription factor blocked *p53*^−/−^*Rfx7*^−/−^ B cell disease trajectory proves its contribution to lymphomagenesis. Moreover, Myc haploinsufficiency provided protection in nonsymtomatic disease, but increased the proportion of mice with symptomatic disease, a phenomenon that might be favored by the converging transcriptional activity on a single *Myc* allele or the reduced Myc-dependent apoptosis^60,61^. Levels of Myc in *Cd19p53*^fl/fl^*Rfx7*^fl/fl^*Myc*^fl/wt^ mice with symptomatic disease were comparable to those of *Cd19p53*^fl/fl^*Rfx7*^fl/fl^ mice, indicating severe deregulation from the functional allele.

Although AID was not strictly required for the aberrant expression of Myc and other pro-tumorigenic genes in *p53*^−/−^*Rfx7*^−/−^ lymphoma, its deletion prevented advanced disease. These results link the oncogenic potential driven by the loss of Rfx7 on *p53*^−/−^ background to AID activity. Corroborating this connection, four out of five cell lines on a *p53*^−/−^*Rfx7*^−/−^ background presented translocations involving the IgH and Myc loci^22,42–44^. Most lymphomas on a *p53*^−/−^*Rfx7*^−/−^ background and in *Iμ-Bcl6Cd19Rfx7*^fl/fl^ mice presented IgM positivity and PB markers, in line with previous observations on *p53*^−/−^ and Bcl6-driven lymphomas^23,62^, with a fraction exhibiting GC features. Despite the limitations of mouse models, these features parallel several aspects of the lymphoid malignancies in which *RFX7* mutations are found^11–17,50,53^.

Taken together, our results support a model whereby the pro-tumorigenic potential of Rfx7-deficient B cells is revealed by ‘second hit’ models. This is in line with the observation that loss of RFX7 in B cells promotes MYC activity, GC B cell and PB expansion. These processes are associated with AID activity^63^, which increases the likelihood of aggressive disease and deregulation of pro-tumorigenic genes in the malignant trajectory. Our results further strengthen the rationale for the use of MYC inhibitors^64^ in lymphomas harboring RFX7 mutations. Deepening our understanding of the mechanisms governing B cell activation, these data further suggest RFX7 relevance for B cell immunity and immune disorders more broadly.

## Methods

### Patient cohort prognosis and *RFX7* mRNA analysis

For the previously published cohorts^49,50^ and normal B cells^66^, we reprocessed the RNA-seq data from raw FASTQ files. Reads were aligned to the GRCh38 reference genome using STAR, and gene-level quantification was performed with featureCounts. For the lymphoma analyses, normalized *RFX7* expression values were incorporated as a continuous covariate in Cox proportional hazards models to evaluate associations with OS and PFS using the survival R package. Furthermore, we employed the survminer R package (https://cran.r-project.org/web/packages/survminer/index.html) to determine the optimal cutoff point for dichotomizing patients into high and low *RFX7* expression groups in terms of OS. Specifically, we used the surv_cutpoint function, which tests all possible expression thresholds iteratively and selects the value that best separates survival outcomes based on the maximally selected rank statistic. This cutoff was then used to define binary groups for OS and PFS outcomes in Kaplan–Meier analysis and Cox modeling. Cell of origin, as described in the relative original publications^49,50^, was used in a multivariate Cox analysis with the stratification induced by *RFX7* expression.

### Mice

*Rfx7*-floxed mice^3^ were crossed to *VaviCre* and R26-stop-EYFP mice (The Jackson Laboratory), *Cd19*Cre^67^ and to *Cγ1Cre* mice^35^ to generate *VavRfx7*^fl/fl^, *Cd19Rfx7*^fl/fl^ and *Cγ1Cre*R26-stop-EYFP*Rfx7*^fl/fl^ mice. Further, *Cd19Rfx7*^fl/fl^ and control *Cd19Rfx7*^wt/wt^ mice were crossed onto mice expressing the *Iμ-Bcl6* transgene^23^ or with floxed *Trp53* gene (The Jackson Laboratory) mice and additionally onto *Myc*-floxed mice^33^ or AID full body knockout mice^22^. All animal experimental protocols were approved by the Veterinary Office regulations of the Cantone Ticino, Switzerland, and all methods were performed in accordance with the Swiss guidelines and regulations. Both male and female mice were used. The mice were housed in individually ventilated cages under standardized conditions (20 ± 2 °C, 55 ± 8% relative humidity, 12 h light/dark cycle) with cage enrichment, and access to food and water ad libitum.

### Survival

*Cd19p53*^fl/fl^, *Cd19p53*^fl/fl^*Rfx7*^fl/fl^, *Cd19p53*^fl/fl^*Rfx7*^fl/fl^*Myc*^fl/fl^*, Cd19p53*^fl/fl^*Rfx7*^fl/fl^*Myc*^fl/wt^ and *Cd19p53*^fl/fl^*Rfx7*^fl/fl^*Aicda*^−/−^ mice were followed by visual inspection and by measuring weight and temperature once per week for 300 days (±7 days). Humane endpoints were based on cumulative scores requiring daily monitoring and consideration of euthanasia if not improved after 1 day when reflecting isolated symptoms or combinations of mild symptoms (including reduced grooming or activity, limited weight or temperature loss) and immediate euthanasia with scores reflecting severe symptoms, such as moribund state, visibly enlarged organs, ≥12% bodyweight loss and temperature lower than 34.5 °C. *Iμ-Bcl6Cd19Rfx7*^fl/fl^ and *Iμ-Bcl6Rfx7*^fl/fl^ mice were inspected visually, and weight was measured every second week from 6 months onwards. Humane endpoints were based on cumulative scores requiring daily monitoring and consideration of euthanasia if not improved after 1 day when reflecting isolated symptoms or combinations of mild symptoms (including reduced grooming or activity, limited weight loss) and immediate euthanasia with cumulative scores reflecting concomitant moderate symptoms or any severe symptom, such as moribund state, abnormal masses and ≥15% bodyweight loss.

### Human lymphoma cell lines

NU-DUL-1 (ACC 579 (ref. ^68^)) B cell lymphoma cell line and CA-46 (ACC 73 (ref. ^69^)) Burkitt lymphoma cell line were purchased from DSM GmbH and maintained in culture according to manufacturer’s instructions using complete medium (RPMI, 10% FBS, 1% penicillin-streptomycin, 1% MEM NEAA, 1% GlutaMAX, 1% sodium pyruvate, 0.1% 2-mercaptoethanol, all from Gibco) as well as U-2932 (DLBCL, provided by F. Bertoni, Bellinzona). Whole-cell lysates were prepared using E1A buffer (250 mM NaCl, 1% NP-40, 50 mM Hepes pH 8) and equal amount of material was used in western blots as described in ‘Immunoblot analysis.’

### Lymphoma cell growth in vitro and transplantation

Spleen from healthy controls (age-matched *Cre*-negative littermates) or tumor-bearing mice (increased percentage of blastoid cells) were processed to single-cell suspensions and treated with hypotonic buffer (155 mM NH_4_Cl, 10 mM KHCO3, 0.1 mM Na_2_EDTA) to lyse erythrocytes. Cells were plated at a concentration of 1–2.5 × 10^6^ cells ml^−1^ (in different experiments) in complete medium (RPMI, 10% FBS, 1% penicillin-streptomycin, 1% MEM NEAA, 1% GlutaMAX, 1% sodium pyruvate, 0.1% 2-mercaptoethanol, all from Gibco). Cells were split according to density and grown for a period of at least 2 weeks. Survival of cells was checked by flow cytometry staining using propridium iodide (ImmunoChemistry Technologies) and cells expanding for at least 2 weeks were considered ‘lines.’ For testing transplantability, 1 × 10^6^ lymphoma cells from the established cell lines were injected subcutaneously into the flank of C57BL/6 recipient mice. Tumor size was measured regularly and the mice were euthanized 10–13 days post-transplantation, when the tumor was between 100 mm^3^ and 600 mm^3^ (maximal tumor size permitted was 1,000 mm^3^). Humane endpoints were based on cumulative scores requiring daily monitoring and consideration of euthanasia when mice presented moderate symptoms (including reduced activity, slightly worsened body condition) and immediate euthanasia with symptoms such as moribund state, tumor size more than 1,000 mm^3^ and worsened body condition.

### Translocation PCR-based analyses

Total genomic DNA from mouse lymphoma B cell lines was extracted using the QIAamp Blood Mini Kit (Qiagen). For each cell line, 500 ng of genomic DNA, corresponding to 10^5^ cells, was used as a template for PCR amplification. Nested PCR was performed using the Expand Long Template PCR System (Roche) with primers described by Ramiro and colleagues^70^. Briefly, for derivative chromosome (chr.) 12 translocations, the first PCR used the following primers: 5′-TGAGGACCAGAGAGGGATAAAAGAGAA-3′ and 5′-GGGGAGGGGGTGTCAAATAATAAGA-3′; the second round of PCR used the following primers: 5′-CACCCTGCTATTTCCTTGTTGCTAC-3′ and 5′-GACACCTCCCTTCTACACTCTAAACCG-3′; for derivative chr. 15 translocation the first round of PCR used the following primers: 5′-GTGAAAACCGACTGTGGCCCTGGAA-3′ and 5′-ACTATGCTATGGACTACTGGGGTCAAG-3′; the second round of PCR used the following primers: 5′-GTGGAGGTGTATGGGGTGTAGAC-3′ and 5′-CCTCAGTCACCGTCTCCTCAGGTA-3′. All primers were used at 300 nM final concentrations. PCR conditions were as follow: 92 °C for 2 min followed by ten cycles of (92 °C 10 s; 60 °C 30 s; 68 °C 7 min) and 25 cycles of (92 °C 10 s; 60 °C 30 s; 68 °C 7 min (with an additional extension time of 20 s per cycle)) for the first round. A 1 μl aliquot of the first round PCR product was used in the second round with the following cycling conditions: 92 °C for 2 min followed by ten cycles of (92 °C 10 s; 60 °C 30 s; 68 °C 7 min) and 19 cycles of (92 °C 10 s; 60 °C 30 s; 68 °C 7 min, with an additional extension time of 20 s per cycle). PCR products were analyzed on a 0.8% agarose gel. DNA corresponding to the band of interest was extracted from the agarose using the QIAquick Gel Extraction KIT (Qiagen) and sent for sequencing (ONT sequencing Microsynth). Sequences were analyzed by mapping the results to the mouse genome (GRCm39).

### Immunization

Mice were immunized with 100 µg (4-hydroxy-3-nitrophenylacetyl–keyhole limpet hemocyanin (NP–KLH) (Biosearch Technologies), precipitated with 0.6 mg Imject-alum (Thermo Scientific) in PBS. Mice were injected intraperitoneally and analyzed 10 days later.

### B cell activation

B cells were isolated from the spleen using EasySep Mouse B cell Isolation Kit (STEMCELL Technologies). For flow cytometry analysis, FO B cells were further FACS-sorted (SORP FACSymphony S6, BD Biosciences) for B220^+^/CD19^+^/CD93^−^/CD23^+^ and seeded at 100,000 cells per well in 96-flat bottom well plate in complete medium (RPMI, 10% FBS, 1% penicillin-streptomycin, 1% MEM NEAA, 1% GlutaMAX, 1% sodium pyruvate, 0.1% 2-mercaptoethanol, all from Gibco). Cells were activated by CpG (Microsynth) added to a final concentration of 2 µg ml^−1^ or by anti-IgM (Jackson ImmunoResearch), coated overnight at a concentration of 5 μg ml^−1^, together with IL-4 (PeproTech) added to a concentration of 2.5 ng ml^−1^ and cultured at 37 °C for 18 h. For proliferation analysis, sorted FO B cells were labeled with Cell Trace Violet (Thermo Fisher) according to manufacturer’s instructions and seeded and stimulated as above, for 40 h (CpG) or 48 h (IL-4 or anti-IgM + IL-4). For immunoblot analysis, B cells were seeded at 1.3 × 10^6^ cells per well in 24-flat bottom well plate. Cells were then activated by CpG (2 µg ml^−1^) or plate-bound anti-IgM (5 μg ml^−1^) + IL-4 (2.5 ng ml^−1^) and cultured at 37 °C in complete medium for 0 h, 6 h and 18 h.

### Immunoblot analysis

B cells enriched and activated as described above, were washed with ice-cold PBS and lysed in Laemmli buffer (187.5 mM Tris-HCl pH 6.8, 6% SDS, 0.03% Bromophenol Blue, 30% glycerol). Cell lysates were boiled for 5 min at 95 °C and separated on a precast (4–20%) gradient SDS–PAGE gel (GenScript) run in MOPS buffer. Proteins were then transferred onto nitrocellulose membranes. Membranes were blocked with 5% milk in PBS 0.1% TWEEN 20 (Sigma) for 15 min at 20–25 °C followed by overnight incubation with the primary antibodies against LDHA (polyclonal), RPL11 (D1P5N), MYC (D84C12), CcnaA2 (E9Q5G), CDC20 (D6C2Q), P53 (D2H90), γH2AX (polyclonal), AID (L7E7), β-ACTIN (E4D9Z), GAPDH (D4C6R), β-TUBULIN (D3U1W) and Histone H3 (D1H2) from Cell Signaling Technology, RFX7 (NBP1-71819) from Novus (1:1,000 dilution), RFX7 (A303-061A) from Bethyl Laboratories (1:1,000 dilution) and VINCULIN (7F9) from Santa Cruz Biotechnology (1:3,000 dilution) in 5% milk in PBS–0.1% TWEEN 20 at +4 °C. Blots were washed with PBS 0.1% TWEEN 20 and incubated with respective HRP-conjugated IgG secondary antibodies from Cell Signaling Technology (1:2,500 dilution) for 30 min at 20–25 °C followed by six rounds of washing and developed using chemiluminescent substrate solution (WesternBright ECL HRP Substrate, Advansta) and imaged by image analyzer machine (Fusion FX, Vilber). Immunoblot images were analyzed using ImageJ software^71^.

### Confocal microscopy

Spleens were fixed in 4% paraformaldehyde overnight at 4 °C and sections (150 μm) were cut with a Leica VT1200S Vibratome. Sections were stained for IgD (11–26c.2a conjugated to Alexa Fluor 594, Biolegend) and GL-7 (GL-7, conjugated to Alexa Fluor 647, Biolegend). Mosaic *Z* stack images were taken with a Leica Stellaris 8 laser scan confocal microscope, with a HC PL APO CS2 ×10/0.40 numerical aperture DRY objective. Excitation was performed with a White Light Laser (excitation lines 590 nm and 653 nm) and collection windows were set to 595–652 nm and 662–829 nm, respectively. Acquisition was set to pinhole 2 a.u. in bidirectional mode, with a pixel size of 570 nm, pixel dwell time of 0.6 µs and line average of 2. Mosaic *Z* stacks of a total size of about 50 µm each with a *Z* step of 5 µm were acquired and then later reconstructed with LAS X software. Images were analyzed using ImageJ software^71^: from Z-stacks, a Maximum Intensity projection was created, minimum, maximum and contrast were set linearly according to control images and then images were overlaid to a final composite and converted to RGB images. Regions of interest were drawn manually.

### Histology and immunohistochemistry

Tissues were fixed in 4% formaldehyde for up to 48 h. The tissues were embedded in paraffin, cut to a thickness of 2 µm and then stained with hematoxylin and eosin (H&E) (Medite cat. no. TST 15). The slides were analyzed at the Istituto Cantonale di Patologia di Locarno, EOC.

For immunohistochemistry (IHC), following paraffin embedding, serial sections (4-μm thick) were obtained using a microtome and incubated at 60 °C for 1 h in a dry oven. Deparaffinization was performed with two consecutive 5-min incubations in OTTIX Plus (Diapath, cat. no. X0076), followed by a 5-min incubation in OTTIX Shaper (Diapath, cat. no. X0096). Heat-induced epitope retrieval was carried out in either citrate buffer (pH 6.0) or Tris-EDTA buffer (pH 9.0), depending on the antibody, at 98 °C for 20 min. Endogenous peroxidase activity was blocked using 3% hydrogen peroxide (VWR Chemicals, cat. no. 23615.248) for 10 min and nonspecific binding was minimized using Protein Block solution (DAKO, Agilent Technologies, cat. no. X0909) for 10 min. Sections were incubated for 1 h at 20–25 °C with primary antibodies against PNA (VectorLabs cat. no. B-1075-5 1:400 pH 6), CD45R/B220 (BioLegend cat. no. RA3-6B2 1:1,000 pH 9) and IRF-4 (CST cat. no. E8H3S 1:600 pH 6) followed by appropriate secondary detection and chromogenic visualization. Slides were counterstained with H&E, dehydrated and coverslipped. Slides were scanned using the Aperio AT2 Slide Scanner (Leica Biosystems) and digital images were analyzed using ImageScope software.

### CDR3-sequencing

Genomic DNA from formalin-fixed and paraffin embedded samples was extracted using DNeasy Blood and Tissue Kit (Qiagen), following the manufacturer’s instructions. Optimal cutting temperature compound-frozen samples were processed and embedded subsequently in paraffin before DNA extraction. For sequencing, libraries were constructed using Ion AmpliSeq Mouse BCR IGH SR Assay (Thermo Fisher Scientific) according to the manufacturer’s instructions, using a DNA input ranging from 50 ng to 100 ng. Template preparation and enrichment were carried out on Ion Chef instrument (Thermo Fisher Scientific) and pooled libraries were sequenced on Ion GeneStudio S5 Prime (Thermo Fisher Scientific) using Ion 550 Chips, yielding a total of 1.3–4.6 million reads per sample.

Sequencing data were analyzed using Ion AmpliSeq Mouse BCR IGH SR (w.1.4) workflow on Ion Reporter software v.5.20.8.0 (Thermo Fisher Scientific). Resulting sequences were analyzed with the online version of IgBLAST using the default alignment options (https://www.ncbi.nlm.nih.gov/igblast/igblast.cgi) to analyze mutations in the CDR3 sequence.

### Flow cytometry

Spleen, BM and iLNs were processed to single-cell suspensions and erythrocyte lysis was performed for spleen and BM with hypotonic buffer (155 mM NH_4_Cl, 10 mM KHCO_3_, 0.1 mM Na_2_EDTA). For flow cytometry analysis, cells were preincubated with anti-CD16/32 (clone 2.4G2) from invivoMab, to block Fc receptors and then surface stained with antibodies against CD19 (6D5), CD21 (7E9), CD23 (B3B4), CD38 (90), CD86 (GL-1), CD93 (AA4.1), CD138 (281-2), B220 (RA3-6B2), IgM (11/41), IgD (11-26.c2a), IgK (187.1), IgL (RML-42) and CXCR4 (L276F12). Antibodies were purchased from BioLegend, Invitrogen and eBioscience, anti-CD95 (Jo2) from BD Biosciences. Dead cells were stained using propidium iodide (ImmunoChemistry Technologies), or Zombie yellow Fixable Viability kit (Biolegend). Staining was performed with appropriate combinations of fluorophores. Intracellular stainings were performed with antibodies against Ki67 (SolA15), Bcl6 (7D1), Irf-4 (IRF-4.3E4), Pax5 (1H9) and Blimp-1 (5E7) purchased from BioLegend and Invitrogen and with c-Myc/n-Myc (cat. no. D3N8F) from Cell Signaling Technology using the Thermo Fisher Scientific Foxp3/Transcription Factor Staining Buffer Set (cat. no. 00-5523-00). After surface staining, cells were fixed with fixation/permeabilization buffer and then further permeabilized according to the manufacturer’s instructions. The intracellular staining antibody was added in permeabilization buffer for 60 min at 20–25 °C. Data were acquired with a Becton Dickinson LSRFortessa flow cytometer and analyzed in FlowJo software (Tree Star).

### Cloning and transfection

The pLenti-ORF clone of human WT RFX7 with a Myc-DDK tag (C-terminal) construct was purchased from OriGene (RC211086L1). The different truncations of RFX7 were generated by PCR using the Q5 High Fidelity polymerase and subcloned (*Bam*HI–*Mlu*I) into pLenti-Myc-DDK vector. The following primers were used: common forward primer (5′-GACTGGATCCGGTACCGAGGA-3′) and distinct reverse primers for each truncation: Q355rev (5′-AAAACGCGTTTGAGGAGAAAGGATTGAAGGATTTCC-3′) Q688rev (5′-AAAACGCGTCTGCCCTTCTATGGTAGCTGCTG-3’) W782rev (5’-AAAACGCGTCCATCCATTTGGATTAAAGCTGCCAAC-3′) Q870rev (5′-AAAACGCGTCTGGGAAACAGAAGGCTCAAACTC-3′). pcDNA3.1(+)-N-term. eGFP-RFX7 WT and mutants and pcDNA3.1(+)-N-term. mCherry-RFX7 WT were outsourced (GenScript cloning service). These constructs contain the full CDS of human *RFX7* bearing a STOP codon immediately after the amino acid Q688 or W782. The NLS mutant contains the following single amino acid mutation: K673A–K674A.

HEK293T cells (from ATCC) were seeded at 2.5 × 10^6^ per 100-mm dish and transfected 24 h later using JetPrime reagent (Polyplus) according to the manufacturer’s instructions always keeping the final amount of DNA to 5 µg. Transfected cells were harvested 24 h post-transfection and FACS-sorted according to eGFP to select transfected cells. Protein expression was verified by immunoblot.

U-2932 cells were transfected using the NEON system (Invitrogen) and the 100-µl gold-coated tips. Briefly, 3 million cells were resuspended in 110 µl of R buffer (resuspension buffer as provided by the Neon Transfection System 100 µl kit) and plasmids encoding the protein of interest were added keeping the amount of final DNA constant (21 µg). Electroporation setting: 1,350 V, 20 ms, two pulses. Immediately after electroporation, cells were kept overnight in a 6-well plates in RPMI antibiotic free medium. Cells were analyzed 24 h post-transfection.

### RT–qPCR

Cell pellets were frozen in TRIzol Reagent (Life technologies) and RNA was extracted according to manufacturer’s instructions. Genomic DNA was removed by treating the RNA with Turbo DNase (Thermo Fisher) before RT, according to the manufacturer’s instructions. In some experiments RNA was extracted using RNeasy Plus Mini Kit (QIAGEN) according to the manufacturer’s instructions. A total of 2 μg of RNA was retrotranscribed using either random primers (Life technologies) or oligo (dT)20 primer (Microsynth). Annealing was performed at 70 °C for 5 min, followed by incubation with M-MLV Retro transcriptase (Promega), RNasin (Promega) and deoxyribonucleotides (Roche) at 40 °C for 10 min, 45 °C for 50 min and 70 °C for 15 min. Primers used for quantitative PCR (qPCR) were reported previously^3^ or described in Supplementary Table 5. qPCR was performed using SYBR GREEN based master mix (PerfeCTa SYBR Green SuperMixes and FastMixes; Quantabio).

### Chromatin immunoprecipitation

HEK293T cells were seeded at 2.5 × 10^6^ per 100-mm dish 24 h before transfection with empty plasmid (mock) and eGFP-tagged WT RFX7 or indicated mutants using JetPrime reagent according to manufacturer’s instructions, always keeping the final amount of DNA to 5 µg. Cells were harvested and sorted for eGFP-positivity 24 h later. Chromatin was prepared using the truChIP Chromatin Shearing Kit (Covaris). Briefly, after sorting cells were washed twice in ice-cold PBS (200rfc, 5 min), resuspended in 1.5 ml of fixing Buffer A and 11.1% paraformaldehyde added to reach 1% final concentration. The cell suspension was incubated for 10 min at 20–25 °C and quenched by adding quenching buffer E for 5 min at 20–25 °C. Cells were collected by centrifuging at 500rcf for 5 min at 20–25 °C. The cell pellet was washed twice with ice-cold PBS. Nuclei were prepared following manufacturer’s instructions for high cell numbers. Shearing of the chromatin was performed using 1-ml AFA tubes (Covaris) using Covaris sonicator and following the manufacturer’s instructions. Chromatin was reverse cross-linked and purified using QIAGEN DNA purification columns and quality checked on a 1% agarose gel (fragment range between 200 bp and 1,000 bp). DNA LoBind Eppendorf tubes or Safe Lock Eppendorf tubes were used throughout the process. Sheared chromatin (10 µg) was precleared using 60 µl of Sepharose 6B beads supplemented with Protease Inhibitor (Cocktail 2 SIGMA) and Salmon Sperm DNA (10 mg ml^−1^; Invitrogen) (1:100 dilution) in a final volume of 600 µl for 1 h at 4 °C with rocking. After centrifugation at 5,200*g*, 1% of material was transferred to a clean 1.5-ml tube and used as input. The remaining precleared chromatin was incubated overnight at 4 °C with either 10 µg of anti-GFP antibody (abcam ab290) or Isotype Control antibody (Cell Signaling). The next day, 30 µl per reaction of Protein A beads was added to the mix of chromatin and antibody followed by 90 min of incubation at 20–25 °C with rotation. After this time, beads went through a series of washes (seven to ten washes). Tubes were changed at each buffer change. After the final wash, 110 µl of room temperature (20–25 °C) Elution Buffer (EB: 10 mM Tris-HCl pH 8.0, 5 mM EDTA, 300 mM NaCl, 0.5% SDS—prepared and stored at 20–25 °C after filtering) were added to the beads and to the input material. For each sample a pre-mix of: 6 µl of EB and 2 µl of RNase A (10 mg ml^−1^) was prepared and 8 µl added to each tube. Tubes were incubated at 37 °C for 30 min with shaking. Meanwhile, for each sample a pre-mix of: 3 µl of EB and 2.5 µl of Proteinase K (20 mg ml^−1^) was prepared and 8.5 µl added to the tubes after the 30 min incubation with RNaseA. Tubes were incubated for additional 2 h at 37 °C shaking, followed by overnight incubation at 65 °C. The next day, beads were centrifuged at 5,200*g* and the supernatant transferred to a clean tube. DNA was extracted using the QIAGEN DNA purification columns. qPCR was performed using the SYBR GREEN based master mix (PerfeCTa SYBR Green SuperMixes and FastMixes; Quantabio) and the primers described in Supplementary Table 5.

### Ex vivo and CpG-stimulated B cell and U-2932 cell library preparation and sequencing

FO B cells were FACS-sorted as B220^+^/CD19^+^/CD23^+^ and either processed directly or activated with CpG (2 μg ml^−1^) for 18 h in vitro. Total RNA was extracted from sorted cells with Monarch Total RNA Miniprep Kit (New England Biolabs) according to the manufacturer’s instructions. For U-2932, cells were transfected using the NEON system (Invitrogen) and 100-µl gold-coated tips (three independent transfections). Briefly, three million cells were resuspended in 110 µl of R buffer and plasmids encoding the protein of interest were added keeping the amount of final DNA constant (21 µg). Electroporation setting: 1,350 V, 20 ms, 2 pulses. Immediately after electroporation, cells were seeded in a 6 wells plate in RPMI antibiotic free medium and analyzed 24 h later. Total RNA was extracted using RNeasy Plus Mini KIT (QIAGEN) from double- or single-positive eGFP/mCherry sorted cells according to manufacturers instruction. Quality controls were performed on Bioanalyzer 2100 using RNA Integrity Number (RIN) > 8 (Agilent Technologies) and Qubit V4 (Invitrogen Thermo Fisher Scientific). NEBNext Ultra Directional RNA Library Prep Kit for Illumina (New England BioLabs Inc.) was employed with the NEBNext Multiplex Oligos for Illumina (New England BioLabs Inc.) and NEBNext Poly(A) mRNA Magnetic Isolation Module for cDNA synthesis with the addition of barcode sequences. The sequencing of the pre-pools was performed using the NextSeq2000 (Illumina) with the P2 reagents kit V3 (100 cycles; Illumina). Samples were processed starting from stranded, single-ended 120-bp-long sequencing reads.

### Ex vivo, CpG-stimulated B cell and transfected U-2932 bioinformatic data analysis

Read quality was verified initially using FastQC (https://www.bioinformatics.babraham.ac.uk/index.html) and MultiQC^72^. Reads were trimmed subsequently to remove sequencing adapters using cutadapt v.2.8^73^ or Trimmomatic v.0.39. Estimates of transcript abundance for all transcripts was performed using HISAT2 v.2.1.0^74^ using either the mouse reference genome version GRCm39 or GENCODE (v.GRCh38). HTSeq-count v.2.0.2^75^ was used to generate the table of counts containing the number of reads mapping to each feature in each sequencing sample. Expression levels were calculated as counts per million (CPM). Statistical testing of differential expression of genes was performed using DESeq2 with the Wald test^76^. For mouse data, transcripts were considered differentially expressed when false discovery rate (FDR) < 0.05, whereas for U-2932 transcripts were considered differentially expressed when FDR = 0.05 and log_2_ fold change (FC) = 1. GSEA was performed using the Hallmark gene sets from MSigDB^31^ using fast GSEA (fGSEA)^77^, a geneset was considered significant if *Q* < 0.05. Statistical computation and visualization (heatmap, volcano plot, activation maps) were performed using the Omics Playground v.4.0.4 (ref. ^78^).

### JASPAR analysis

Transcription factor binding sites were analyzed with the JASPAR tool (http://jaspar.genereg.net/)^29^. Promoter sequences were collected in the eukaryotic promoter database (https://epd.expasy.org/epd/), to span −700 to +300 bp over the described transcription start site of each gene. A relative profile score threshold of 85% was used to screen for similarities with the matrix profile MA1554.1 described in the JASPAR CORE database. Screened promoters belong to the control, the Rfx7-target and the E2F upregulated gene groups. The latter corresponds to the top 20 upregulated genes of the E2F signature^29^ in CpG-activated Rfx7-deficient B cells based on our RNA-seq data (*Stmn1*, *Ccnb2*, *Cdca3*, *Birc5*, *Plk1*, *Kif22*, *Tcf19*, *Hmmr*, *Cdk1*, *Cdca8*, *Cks1b*, *Tk1*, *Rrm2*, *Tacc3*, *Ccne1*, *Aurka*, *Cdc20*, *Rad51c*, *Spag5* and *Mxd3*). The control group corresponds to 34 nonmodulated genes (log_2_FC > −0.018 and <0.024; meta.*Q* > 0.99, chosen randomly) selected from the list of genes from our RNA-seq (*Adamdec1, Btnl6, C1qa, C6, Ccr3, Cd14, Cd200r1, Cd276, Cd300a, Cd55b, Clec4n*, *Clec7a, Clu, Cxcl9, Dysf, Ephx2, Fcgr1, Fpr1, Gad1, Hcar2, Hpgd, Hs3st2, Ifit3b, Kctd12b, Loxhd1, Lsmem1, Mafb, Paqr9, Pf4, Pilrb2, Pparg, Siglec1, Slco2b1* and *Tlr8*). The 42 Rfx7-targets were selected among RFX7 targets identified by ChIP^5^ and found to be tendentially decreased (log_2_FC < −0.2) in the current or previously published mouse RNA-seq data^3^ (*Dip2a*, *Klf9*, *Ptms*, *Tob2*, *Arl15*, *Dolpp1*, *Maf*, *Top2b*, *Atrx*, *Mxd4*, *Rap2a*, *Cabin1*, *Emc9*, *Rev3l*, *Tsc22d1, Rfx5, Ccnd1*, *Fam214a*, *Pdcd4*, *Rmnd5a*, *Tspyl2*, *Ccng2*, *Hnrnpul2*, *Ube2h*, *Cdk4*, *Pik3ip1*, *Sesn3*, *Cic*, *Ip6k2*, *Slc43a2*, *Ypel2*, *Junb, Plcxd2*, *Sox12, Cks2*, *Kdm4a*, *Pnrc1*, *Sox4, Ddit4*, *Kdm6b*, *Prkcz* and *Tob1*).

### Protein structural prediction of truncated and full-length RFX7

The full-length structure of human RFX7 (UniProt ID Q2KHR2) was predicted using the Boltz-1 server^79^, which implements an AlphaFold3-based algorithm for protein structure prediction. The sequence of RFX7 was retrieved from the UniProt database and used as input. The prediction was performed including DNA as a ligand and using the X-ray structure of hRFX1 DNA-binding domain (PDB 1DP7) as template for the RFX7 DNA-binding domain. The structural models of the RFX7 forms ending at residue Q688 (RFX7 1–688) and at residue W782 (RFX7 1–782) were generated using SwissModel^80^, using the full-length RFX7 model predicted by Boltz-1 as the template.

The structural stability of the predicted RFX7 1–688 and RFX7 1–782 models was evaluated using molecular dynamics simulations performed with GROMACS 2019 (v.2019.6). Each model was solvated in a TIP3P water box, neutralized with counterions, and energy-minimized before simulation. Production molecular dynamics runs were carried out under NPT conditions for 100 ns at 300 K using the AMBER99SB-ILDN force field^81^. Trajectory analysis, including root-mean-square deviation, was performed to assess model stability.

### Statistical analysis

Statistical analyses were performed using Prism software (GraphPad v.5.0). Statistical tests are described in the figure legends. Outliers present in the dataset of Fig. 3b (and Extended Data Fig. 3c) were analyzed according to the literature^82^. After graphing outliers and checking with normal probability plots, we excluded normality of data. Thus, we used the interquartile range method to create outlier fences. Once outliers have been identified, we checked that the percentage was below a standard limit (such as 5% in trimmed mean) and we excluded those values, being confident of noninformativeness of outliers.

### Reporting summary

Further information on research design is available in the Nature Portfolio Reporting Summary linked to this article.

## Online content

Any methods, additional references, Nature Portfolio reporting summaries, source data, extended data, supplementary information, acknowledgements, peer review information; details of author contributions and competing interests; and statements of data and code availability are available at https://doi.org/10.1038/s41590-026-02526-2.

## Supplementary information

Supplementary Information Legends for Supplementary Tables 1–5.

Reporting Summary

Supplementary Table 1 **a**, CDR3-sequencing of genomic DNA extracted from the six fixed spleens from 7- to 8-month-old *Iμ-Bcl6Cd19Rfx7*^fl/fl^ mice. Productive clones were screened for somatic mutations. **b**, CDR3-sequencing of genomic DNA extracted from cell lines, from enriched control B cells, or fixed spleens from symptomatic *Cd19p53*^fl/fl^*Rfx7*^fl/fl^*Myc*^wt/fl^ mice. Productive clones (frequency >1.5%) were screened for somatic mutations.

Supplementary Table 2 List of the DEGs between nonstimulated or CpG-stimulated *Rfx7*^−/−^ and control FO B cells. The table shows the list of DEGs with Ensemble entry ID, gene acronym and full name, chromosome number, log_2_FC, average log_2_ CPM expression (logCPM, AveExpr) and metaq value.

Supplementary Table 3 **a**, *RFX7* mutations identified in the indicated tumor types and studies, and information on the status of *TP53*, *BCL6* and *MYC* in *RFX7*-mutated tumor samples. **b**, Frequency of *RFX7* mutations in the cohorts analyzed and co-occurrence of *TP53* alterations.

Supplementary Table 4 List of the DEGs between the indicated conditions in transfected U-2932 cells. The tables show the list of DEGs with Ensemble entry ID, gene acronym and full name, chromosome number, log_2_FC, average log_2_ CPM expression (logCPM, AveExpr), metaq value and deseq2.wald.q value.

Supplementary Table 5 List of primers used.

## Source data

Source Data Fig. 1 Statistical source data.

Source Data Fig. 2 Statistical source data.

Source Data Fig. 3 Statistical source data.

Source Data Fig. 4 Statistical source data.

Source Data Fig. 5 Statistical source data.

Source Data Fig. 6 Statistical source data.

Source Data Fig. 2 Unprocessed western blots.

Source Data Fig. 6 Unprocessed western blots.

Source Data Extended Data Fig. 1 Statistical source data.

Source Data Extended Data Fig. 2 Statistical source data.

Source Data Extended Data Fig. 3 Statistical source data.

Source Data Extended Data Fig. 4 Statistical source data.

Source Data Extended Data Fig. 5 Statistical source data.

Source Data Extended Data Fig. 6 Statistical source data.

Source Data Extended Data Fig. 1 Unprocessed western blots.

Source Data Extended Data Fig. 2 Unprocessed western blots.

Source Data Extended Data Fig. 3 Unprocessed western blots.

Source Data Extended Data Fig. 4 Unprocessed western blots.

Source Data Extended Data Fig. 5 Unprocessed western blots.

Source Data Extended Data Fig. 6 Unprocessed western blots.

## Data Availability

The datasets presented in Figs. 2 and 6 are available via GSE308503. The data that support the findings of this study are available from the corresponding author upon reasonable request. Limited-access datasets were obtained from BC CANCER, the European Genome-phenome Archive (dataset identifiers EGAD00001005105, EGAD00001004142 and EGAS00001002657), the National Cancer Institute’s Genome Data Commons Publication Page (https://gdc.cancer.gov/about-data/publications/CGCI-BLGSP-2022-1), and dbGaP under accession numbers phs000450.v3.p1, phs000450.v5.p1 and phs003023.v1.p1. All data were used according to the data use agreements. Source data are provided with this paper.
