# Supplementary material for: Regulatory factor X 7 limits Myc activity during B cell activation and suppresses Myc-dependent lymphomagenesis
Source: Nat Immunol. 2026 Jun 1;27(7):1490–501. doi: 10.1038/s41590-026-02526-2 (PMC13652738; doi:10.1038/s41590-026-02526-2)
Supplement: Supplementary file 1 [file 41590_2026_2526_MOESM14_ESM.pdf]

Fig. 2b

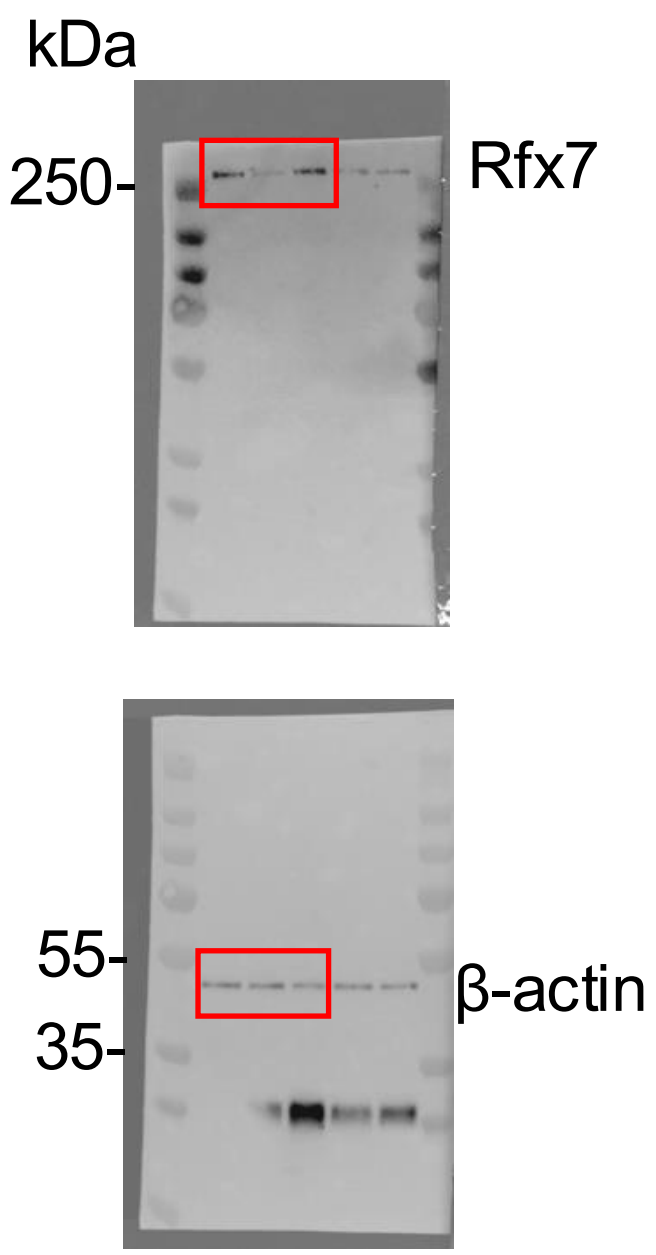

Fig. 2f

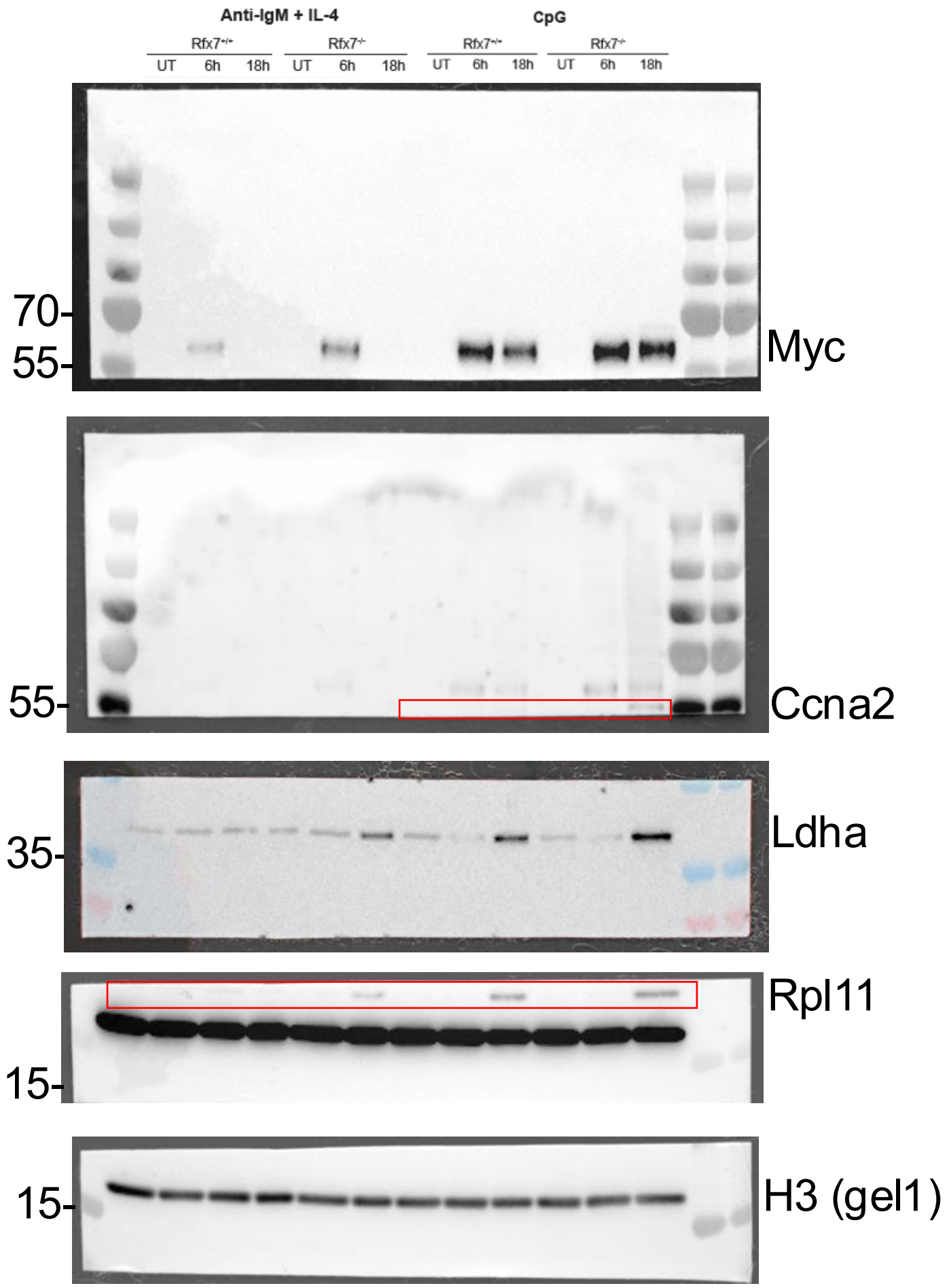

Anti-IgM + IL-4
CpG
UT
6h
18h
70-
55-
Myc
55-
Ccna2
35-
Ldha
Rpl11
15-
15-
H3 (gel1)

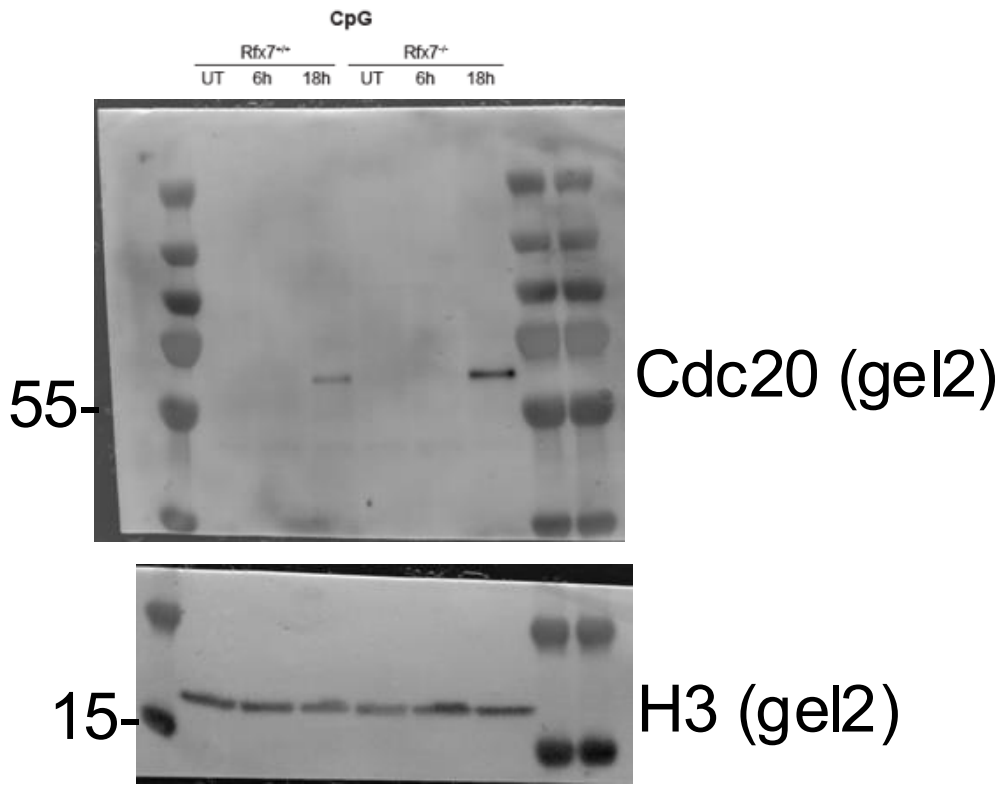

CpG
55-
Cdc20 (gel2)
15-
H3 (gel2)
