# Supplementary material for: Regulatory factor X 7 limits Myc activity during B cell activation and suppresses Myc-dependent lymphomagenesis
Source: Nat Immunol. 2026 Jun 1;27(7):1490–501. doi: 10.1038/s41590-026-02526-2 (PMC13652738; doi:10.1038/s41590-026-02526-2)

Fig. 6c

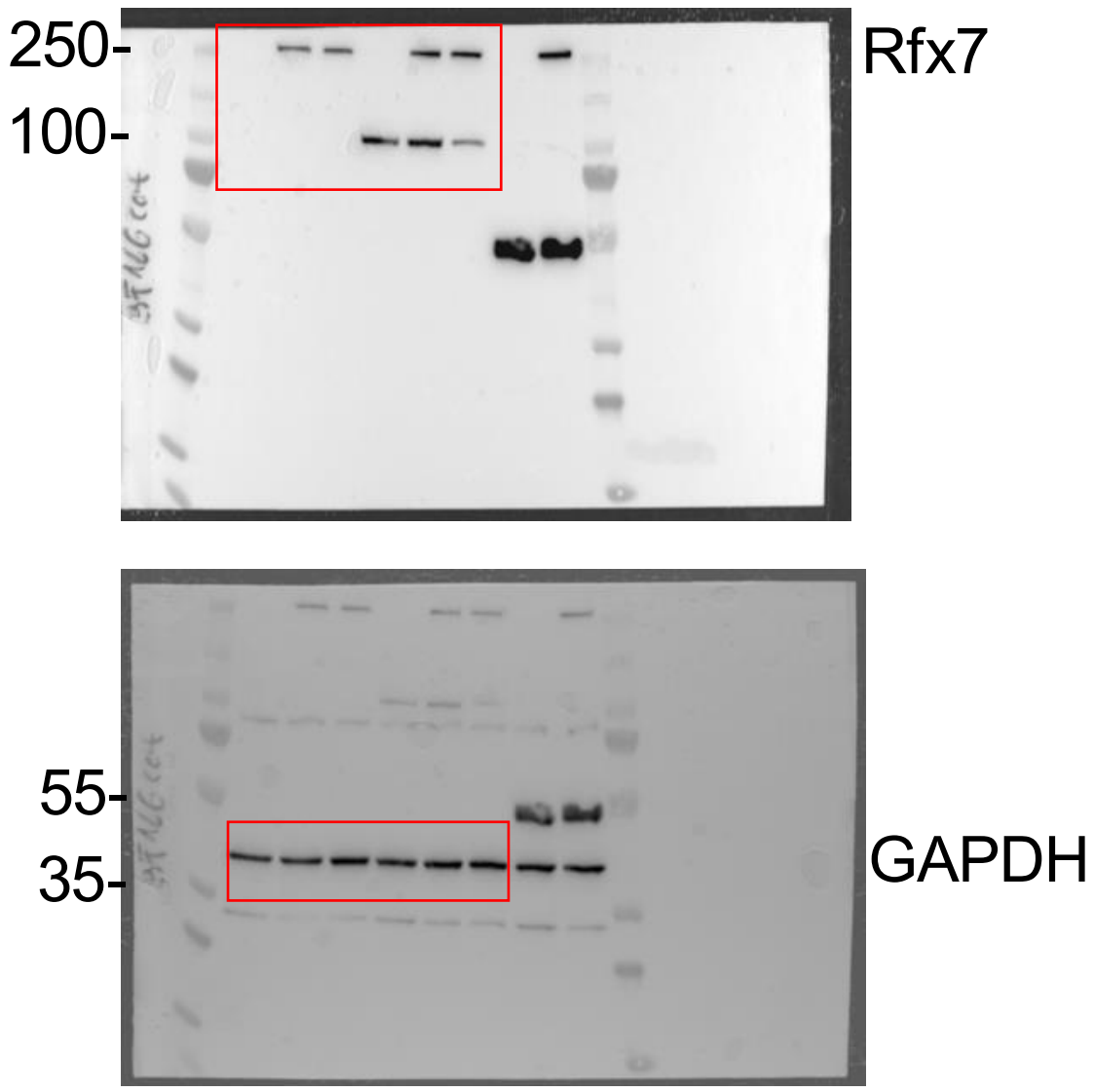

Fig. 6g

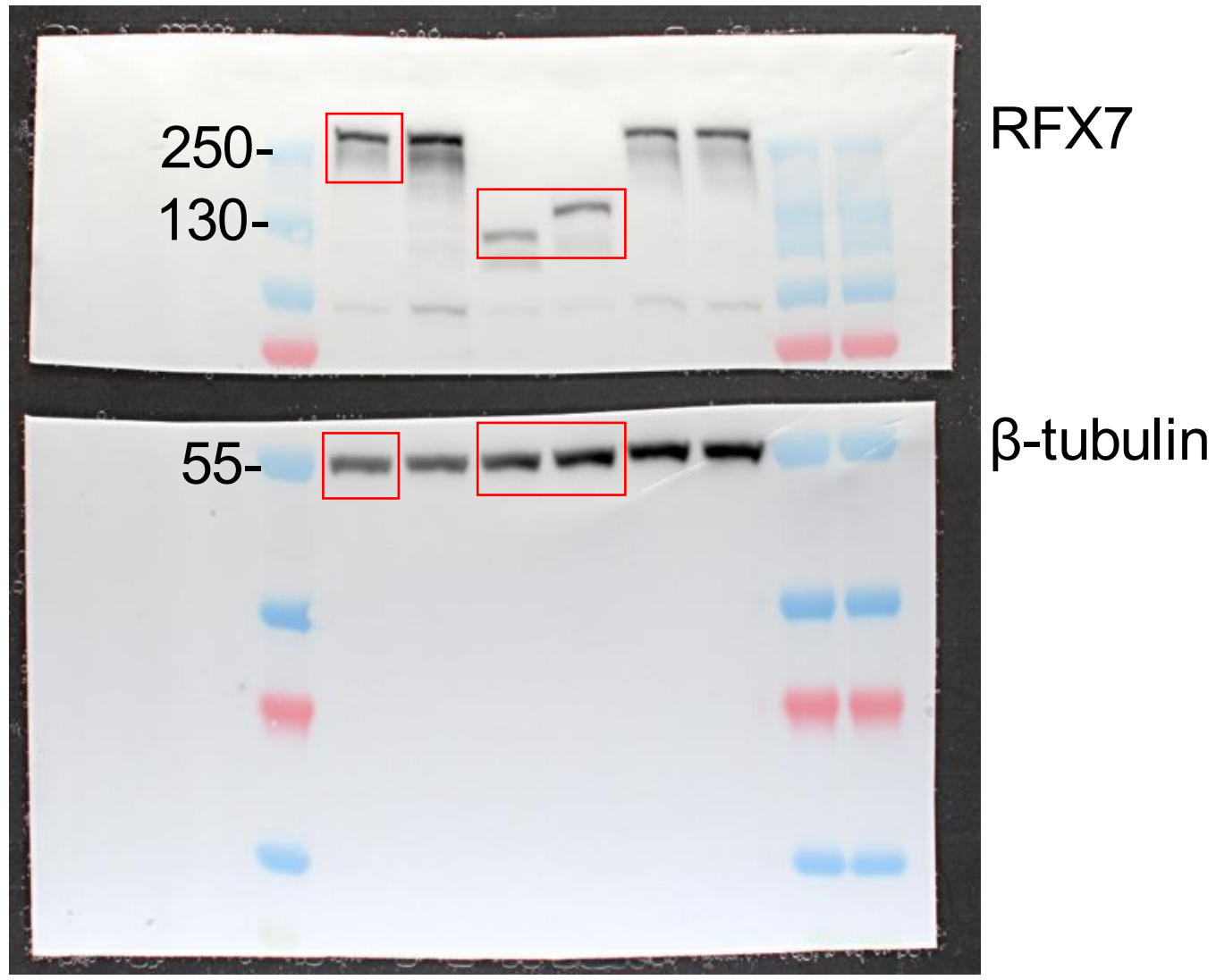

Supplement: Supplementary file 2 [file 41590_2026_2526_MOESM15_ESM.pdf]
