# Supplementary material for: Regulatory factor X 7 limits Myc activity during B cell activation and suppresses Myc-dependent lymphomagenesis
Source: Nat Immunol. 2026 Jun 1;27(7):1490–501. doi: 10.1038/s41590-026-02526-2 (PMC13652738; doi:10.1038/s41590-026-02526-2)
Supplement: Supplementary file 3 [file 41590_2026_2526_MOESM1_ESM.pdf]

nature immunology

Supplementary information https://doi.org/10.1038/s41590-026-02526-2

Regulatory factor X 7 limits Myc activity during B cell activation and suppresses Myc-dependent lymphomagenesis

In the format provided by the authors and unedited

Supplementary File

Supplementary Table Legends

Supplementary Table 1. a) CDR3-sequencing of genomic DNA extracted from the 6 fixed spleens from 7 to 8-month-old *Iμ-Bcl6Cd19Rfx7*$^{fl/fl}$ mice. Productive clones were screened for somatic mutations. b) CDR3-sequencing of genomic DNA extracted from cell lines, from enriched control B cells, or fixed spleens from symptomatic *Cd19p53*$^{fl/fl}$*Rfx7*$^{fl/fl}$*Myc*$^{wt/fl}$ mice. Productive clones (frequency > 1.5%) were screened for somatic mutations.

Supplementary Table 2. List of the DEGs between non-stimulated or CpG-stimulated Rfx7$^{–/–}$ and control FO B cells. The table shows the list of DEGs with Ensemble entry ID, gene acronym and full name, chromosome number, log2 fold change (log2FC), average log2 counts-per-million expression (logCPM, AveExpr), and metaq value.

Supplementary Table 3. a) *RFX7* mutations identified in the indicated tumor types and studies, and information on the status of *TP53*, *BCL6*, and *MYC* in *RFX7*-mutated tumor samples. b) Frequency of *RFX7* mutations in the analysed cohorts and co-occurrence of *TP53* alterations.

Supplementary Table 4. List of the DEGs between the indicated conditions in transfected U-2932 cells. The tables show the list of DEGs with Ensemble entry ID, gene acronym and full name, chromosome number, log2 fold change (log2FC), average log2 counts-per-million expression (logCPM, AveExpr), metaq value, and deseq2.wald.q value.

Supplementary Table 5. List of primers used.
