# Supplementary material for: Regulatory factor X 7 limits Myc activity during B cell activation and suppresses Myc-dependent lymphomagenesis
Source: Nat Immunol. 2026 Jun 1;27(7):1490–501. doi: 10.1038/s41590-026-02526-2 (PMC13652738; doi:10.1038/s41590-026-02526-2)
Supplement: Supplementary file 9 [file 41590_2026_2526_MOESM2_ESM.pdf]

nature portfolio

Corresponding author(s): Greta Guarda

Last updated by author(s): Apr 9, 2026

Reporting Summary

Nature Portfolio wishes to improve the reproducibility of the work that we publish. This form provides structure for consistency and transparency in reporting. For further information on Nature Portfolio policies, see our Editorial Policies and the Editorial Policy Checklist.

Statistics

For all statistical analyses, confirm that the following items are present in the figure legend, table legend, main text, or Methods section.

n/a	Confirmed	
☐	☒	The exact sample size (*n*) for each experimental group/condition, given as a discrete number and unit of measurement
☐	☒	A statement on whether measurements were taken from distinct samples or whether the same sample was measured repeatedly
☐	☒	The statistical test(s) used AND whether they are one- or two-sided<br>*Only common tests should be described solely by name; describe more complex techniques in the Methods section.*
☐	☒	A description of all covariates tested
☐	☒	A description of any assumptions or corrections, such as tests of normality and adjustment for multiple comparisons
☐	☒	A full description of the statistical parameters including central tendency (e.g. means) or other basic estimates (e.g. regression coefficient) AND variation (e.g. standard deviation) or associated estimates of uncertainty (e.g. confidence intervals)
☐	☒	For null hypothesis testing, the test statistic (e.g. *F*, *t*, *r*) with confidence intervals, effect sizes, degrees of freedom and *P* value noted<br>*Give P values as exact values whenever suitable.*
☒	☐	For Bayesian analysis, information on the choice of priors and Markov chain Monte Carlo settings
☒	☐	For hierarchical and complex designs, identification of the appropriate level for tests and full reporting of outcomes
☒	☐	Estimates of effect sizes (e.g. Cohen's *d*, Pearson's *r*), indicating how they were calculated

Our web collection on statistics for biologists contains articles on many of the points above.

Software and code

Policy information about availability of computer code

Data collection	Flow cytometry data was acquired using FACSDiva v9.0.1 on LSRFortessa or FACSymphony™ A5 (BD Biosciences).<br>Cell sorting was performed using FACSDiva v9.0.1 on Sorter Aria (BD Biosciences).<br>Western blot images were acquired using Fusion FX Edge 18.12 on Fusion FX (Vilber).<br>Histology images were acquired on Zeiss Axioskop 2 plus.<br>Confocal images were acquired using Leica LAS X 4.8.5 onLeica Stellaris 8 laser scan confocal microscope.<br>Immunohistochemistry slides were scanned using the Aperio AT2 Slide Scanner (Leica Biosystems).<br>RNA-seq libraries were quantified using a Bioanalyzer 2100 using RNA Integrity Number (RIN) > 8 (Agilent Technologies, Santa Clara, CA, USA) and Qubit V4 (Invitrogen Thermo Fisher Scientific, Waltham, MA, USA). The sequencing of the pre-pools was performed using th NextSeq2000 (Illumina, San Diego, CA, USA) with the P2 reagents kit V3 (100cycles; Illumina).<br>Quantitative Real Time PCR data were acquired using Quantstudio Design & Analaysis software V1.5.3 on Quantstudio 3 PCR machine (Applied Biosystems).

Data analysis

FACS data were analyzed with FlowJo versionS 9 and 10.10.0.
Graphpad Prism version 10 was used for data analysis and statistics.
Omics Playground (v4.0.4+master251127) was used for RNA-seq data analysis (https://bigomics.ch/omics-playground/).
ImageJ Version 2.1.0/1.53c for western blot and Immunoflourescence image analysis.
Immunohistochemistry digital images were analyzed using ImageScope software.
Integrative Genomic Viewer 2.19.6 (IGV) software was used for RNA seq data analysis for NU-DUL-1, CA46 and patient samples
Boltz-1 server(Wohlwend et al., 2025), which implements an AlphaFold3-based algorithm was used for protein structure prediction
GROMACS 2019 (version 2019.6) was used form molecular dynamics simulation

For manuscripts utilizing custom algorithms or software that are central to the research but not yet described in published literature, software must be made available to editors and reviewers. We strongly encourage code deposition in a community repository (e.g. GitHub). See the Nature Portfolio guidelines for submitting code & software for further information.

Data

Policy information about availability of data

All manuscripts must include a data availability statement. This statement should provide the following information, where applicable:

- Accession codes, unique identifiers, or web links for publicly available datasets
- A description of any restrictions on data availability
- For clinical datasets or third party data, please ensure that the statement adheres to our policy

The datasets related to Figs. 2 and 6 are available in the GEO repository under the accession code GSE308503.
The data that support the findings of this study are available from the corresponding author upon reasonable request.

Research involving human participants, their data, or biological material

Policy information about studies with human participants or human data. See also policy information about sex, gender (identity/presentation), and sexual orientation and race, ethnicity and racism.

Reporting on sex and gender

N/A We reanalyzed either publicly available human data or limited access dataset indicated in the Acknowledgment section.

Reporting on race, ethnicity, or other socially relevant groupings

N/A

Population characteristics

N/A

Recruitment

N/A

Ethics oversight

N/A

Note that full information on the approval of the study protocol must also be provided in the manuscript.

Field-specific reporting

Please select the one below that is the best fit for your research. If you are not sure, read the appropriate sections before making your selection.

☒ Life sciences ☐ Behavioural & social sciences ☐ Ecological, evolutionary & environmental sciences

For a reference copy of the document with all sections, see nature.com/documents/nr-reporting-summary-flat.pdf

Life sciences study design

All studies must disclose on these points even when the disclosure is negative.

Sample size

No statistical methods were used to predetermine sample size. Sample sizes are indicated in the figure legends. Dealing with exploratory research, sample sizes were chosen based on our preliminary experiments.

Data exclusions

No data exclusion was applied except in the following cases:
1-Technical issues: data were not considered unless all quality controls included in the experiment were met
2-For quantitative PCR/RT-PCR, outlier values within triplicates were excluded (cf. Current protocols in molecular biology)
3-Outliers present in the data set of Figure 3B (and Extended Data Figure 3C) have been analyzed according to the literature. After graphing outliers and checking with normal probability plots, we excluded normality of data. Thus, we used the Interquartile range method to create outlier fences. Once outliers have been identified, we checked that the percentage was below a standard limit (like 5% in trimmed mean) and we excluded those values, being confident of non-informativeness of outliers.

Replication

The number of biological replicates and independent experiments were included in Figure legends.

Randomization

Randomisation was not relevant for our purpose. As we aimed at identifying the function of Rfx7, all experimental groups were constituted according to the genotypes of the animals or cells (e.g. deficient /sufficient).

Blinding	N/A. Blinding was not relevant for our purposes.

Reporting for specific materials, systems and methods

We require information from authors about some types of materials, experimental systems and methods used in many studies. Here, indicate whether each material, system or method listed is relevant to your study. If you are not sure if a list item applies to your research, read the appropriate section before selecting a response.

Materials & experimental systems

n/a	Involved in the study
☐	☒ Antibodies
☐	☒ Eukaryotic cell lines
☒	☐ Palaeontology and archaeology
☐	☒ Animals and other organisms
☐	☒ Clinical data
☒	☐ Dual use research of concern
☒	☐ Plants

Methods

n/a	Involved in the study
☒	☐ ChIP-seq
☐	☒ Flow cytometry
☒	☐ MRI-based neuroimaging

Antibodies

Antibodies used

FACS Abs:
Anti-mouse CD16/32 Clone: 2.4G2 Cat #BE0307 Lot #825322M1 InVivo MAb (1:1500)
Anti-mouse CD19 PE/Cyanine7 Clone: 6D5 Cat #115520 Lot #B368446 Biolegend (1:200)
Anti-mouse CD19 APC/Cyanine7 Clone: 6D5 Cat #115530 Lot #B304959 Biolegend (1:200)
Anti-mouse CD21/35 (CR2/CR1) Pacific Blue Clone: 7E9 Cat #123414 Lot #B420618 Biolegend (1:100)
Anti-mouse CD23 APC/Cyanine7 Clone: B3B4 Cat #101630 Lot #B374855 Biolegend (1:100)
Anti-mouse CD38 Pacific Blue Clone: 90 Cat #102719 Lot #B396843 Biolegend (1:200)
Anti-mouse CD86 APC Clone: GL-1 Cat #105012 Lot #B372258 Biolegend (1:100)
Anti-mouse CD93 (AA4.1, early B lineage) APC Clone: AA4.1 Cat #136510 Lot #B385863 Biolegend (1:200)
Anti-mouse CD138 (Syndecan-1) Brilliant violet 711TM Clone: 281-2 Cat #142519 Lot #B397189 Biolegend (1:200)
Anti-mouse/human CD45R/B220 Brilliant violet 605TM Clone: RA3-6B2 Cat #103243 Lot #B421228 Biolegend (1:200)
Anti-mouse IgM PE Clone: RMM-1 Cat #406508 Lot #B3864149 Biolegend (1:200)
Anti-mouse IgM APC Clone: 11/41 Cat #17-5790-82 Lot #2171317 eBioscience Invitrogen (1:200)
Anti-mouse IgD PE Clone: 11-26c.2a Cat #405706 Lot #B291450 Biolegend (1:200)
Anti-mouse IgD Alexa Fluor 594 Clone: 11-26c.2a Cat #405740 Lot #B335124 Biolegend (1:200)
Anti-mouse IgK FITC Clone: 187.1 Cat #MKAPPA01 Lot #D2116-VF27E Invitrogen (1:200)
Anti-mouse IgL Biotin Clone: RML-42 Cat #407304 Lot # B326197 Biolegend (1:200)
Anti-mouse CD184 (CXCR4) PE Clone: L276F12 Cat #146505 Lot # B370600 Biolegend (1:200)
Anti-mouse/rat Ki-67 FITC Clone: SolA15 Cat #11-5698-82 Lot # 2565715 eBioscience Invitrogen (1:50)
Anti-mouse/human Bcl-6 PE/Dazzle TM 594 Clone: 7D1 Cat #358510 Lot #B314123 Biolegend (1:50)
Anti-IRF4 Alexa Fluor 594 Clone: IRF4.3E4 Cat #646410 Lot #400826 Biolegend (1:50)
Anti-Pax5 PE Clone: 1H9 Cat #649707 Lot #B362582 Biolegend (1:50)
Anti-mouse Blimp-1 APC Clone: 5E7 Cat #150008 Lot #B364127 Biolegend (1:50)
Streptavidin Brilliant violet 711TM Cat #405241 Lot #B373721 Biolegend (1:200)
Anti-mouse CD95 PE/Cyanine7 Clone: Jo2 Cat #557653 Lot #3320907 BD Biosciences (1:100)
c-Myc/N-Myc PE Clone: D3N8F Cat #35876S Lot #2 Cell Signaling Technologies (1:50)

ChIP Abs:
Anti-GFP (ab290) Cat #1037873-7 Abcam (10ug of antibody for 10ug of chromatin)

WB Abs:
Anti-GFP (D5.1) Cat #2956S Lot #6 Cell Signaling Technology (1:1000)
LDHA (polyclonal) Cat #2012S Lot #2 Cell Signaling Technology (1:1000)
AID (L7E7) Cat #4975 Lot#1 Cell Signaling Technology (1:1000)
RPL11 (D1P5N) Cat #18163S Lot #1 Cell Signaling Technology (1:1000)
MYC (D84C12) Cat #56055 Lot #12 Cell Signaling Technology (1:1000)
CcnaA2 (E9Q5G) Cat #81754S Lot #1 Cell Signaling Technology (1:1000)
CDC20 (D6C2Q) Cat #14866S Lot #1 Cell Signaling Technology (1:1000)
P53 (D2H90) Cat# 32532 Lot#2 Cell Signaling Technology (1:1000)
Phospho-Histone H2A.X (Ser139) Cat#2577 Lot#4 Cell Signaling Technology (1:1000)
GAPDH (D4C6R) Cat #97166S Lot #6 Cell Signaling Technology (1:1000)
Histone H3 (D1H2) Cat #4499 Lot #2 Cell Signaling Technology (1:1000)
β-Actin (E4D9Z) Cat# 58169 Lot# 1 Cell Signaling Technology (1:1000)
β-Tubulin (D3U1W) Cat# 86298 Lot# 3 Cell Signaling Technology (1:1000)
Vinculin (7F9) Cat #sc-73614 Santa Cruz Biotechnology (1:3000)
Anti-mouse HRP-conjugated IgG secondary antibodies Cat #77076S Lot #39 Cell Signaling Technology (1:2500)
Anti-rabbit HRP-conjugated IgG secondary antibodies Cat #7074S Lot #33 Cell Signaling Technology (1:2500)
RFX7 (polyclonal) Cat #NBP1-71819 Lot#A1 Novus (1:2000)

	RFX7 Cat # 303-061ALot#1 Bethyl Laboratrories (1:1000)<br><br>Immunohistochemistry Abs:<br>Peanut Agglutinin (PNA) Biotinylated Cat# B-1075-5 VectorLabs (1:400)<br>Anti-mouse/human CD45R/B220 Alexa Flour 488 (RA3-6B2) Cat#103228 Biolegend (1:1000)<br>IRF-4 (E8H3S) Cat #62834 Cell Signaling Technology (1:600)<br><br>Confocal Microscopy Abs:<br>Anti-mouse IgD Alexa Fluor 594 Clone: 11-26c.2a Cat# 2565572 Biolegend (1:100)<br>Anti-mouse/human GL-7 Alexa Fluor 647 Clone: GL-7 Cat# 2562185 Biolegend (1:100)
Validation	The antibodies used in the study were validated by the corresponding manufacturers. Commercial antibodies were used in accordance with the manufactures' recommendation as provided on their official websites or the suitable dilutions were established in our lab by testing.

Eukaryotic cell lines

Policy information about cell lines and Sex and Gender in Research

Cell line source(s)	Human embryonic kidney cell line (HEK293T) was obtained from ATCC (Manassas, VA, USA).<br>NU-DUL-1 (ACC 579 ref) B-cell lymphoma cell line and CA-46 (ACC 73 ref) Burkitt lymphoma cell line were purchased from DSM GmbH.<br>U2932 cell line was provided by Prof. Francesco Bertoni, Institute for Oncology research (IOR), Bellinzona, and were not authenticated by us.<br>Murine cell lines were generated in-house from the tumors as described in Material and Methods. Cell lines 1,4 and 5 were derived from female, and cell lines 2 and 3 were derived from male mice.
Authentication	HEK293T cells were directly obtained commercially from ATCC where these are routinely authenticated.
Mycoplasma contamination	HEK293T and U2932 cells tested negative for Mycoplasma. The murine cell lines generated in house from the spleen tumors were not tested for mycoplasma as well as the NU-DUL-1 and CA46 cell lines.
Commonly misidentified lines<br>(See ICLAC register)	No commonly misidentified lines were used.

Animals and other research organisms

Policy information about studies involving animals; ARRIVE guidelines recommended for reporting animal research, and Sex and Gender in Research

Laboratory animals	Rfx7-floxed mice were crossed to VaviCre and R26-stop-EYFP mice, Cd19Cre and Cγ1Cre mice to generate VavRfx7fl/fl, Cd19Rfx7fl/fl, and Cγ1CreR26-stop-EYFPRfx7fl/fl mice. Further, Cd19 Rfx7fl/fl and control Cd19 Rfx7wt/wt mice were crossed onto mice expressing the Iμ-Bcl6 transgene or with floxed Trp53 gene mice and additionally onto Myc-floxed mice or AID full body knockout mice. The respective references for the above-mentioned strains are listed in material and methods. The mice were housed in individually ventilated cages under standardized conditions (20±2°C, 55±8% relative humidity, 12 h light/dark cycle) with cage enrichment, and access to food and water ad libitum. All mice were generated or backcrossed multiple times on a C57BL/6 background.
Wild animals	N/A
Reporting on sex	Animal experiments do not only apply to one sex. Sex of the animals was considered as part of assigning experimental groups and we tried to represent both sexes across experiments in so far as possible based on availability.
Field-collected samples	N/A
Ethics oversight	All animal experimental protocols were approved by the Veterinary Office regulations of the Cantone Ticino, Switzerland, and all methods were performed in accordance with the Swiss guidelines and regulations.

Note that full information on the approval of the study protocol must also be provided in the manuscript.

Clinical data

Policy information about clinical studies

All manuscripts should comply with the ICMJE guidelines for publication of clinical research and a completed CONSORT checklist must be included with all submissions.

Clinical trial registration	*Provide the trial registration number from ClinicalTrials.gov or an equivalent agency.*
Study protocol	*Note where the full trial protocol can be accessed OR if not available, explain why.*

Data collection	*Describe the settings and locales of data collection, noting the time periods of recruitment and data collection.*
Outcomes	*Describe how you pre-defined primary and secondary outcome measures and how you assessed these measures.*

Plants

Seed stocks	N/A
Novel plant genotypes	N/A
Authentication	N/A

Flow Cytometry

Plots

Confirm that:

☐ The axis labels state the marker and fluorochrome used (e.g. CD4-FITC).

☒ The axis scales are clearly visible. Include numbers along axes only for bottom left plot of group (a 'group' is an analysis of identical markers).

☐ All plots are contour plots with outliers or pseudocolor plots.

☒ A numerical value for number of cells or percentage (with statistics) is provided.

Methodology

Sample preparation	Spleen, bone marrow, and inguinal LNs were processed to single cell suspensions and erythrocyte lysis was performed for spleen and BM with hypotonic buffer (155 mM NH_4Cl, 10 mM $KHCO_3$, 0.1 mM Na_2EDTA). For flow cytometry analysis, cells were preincubated with anti-CD16/32 (clone 2.4G2) to block Fc receptors and then surface stained with antibodies against CD19 (6D5), CD21 (7E9), CD23 (B3B4), CD38 (90), CD86 (GL-1), CD93 (AA4.1), CD138 (281-2), B220 (RA3-6B2), IgM (11/41), IgD (11-26.c2a), IgK (187.1), IgL (RML-42), CXCR4 (L276F12). Antibodies were purchased from BioLegend and Invitrogen, anti-CD95 (Jo2) from BD Biosciences. Dead cells were stained using propidium iodide (ImmunoChemistry Technologies), or Zombie yellow Fixable Viability kit (Biolegend). Staining was performed with appropriate combinations of fluorophores. Intracellular stainings were performed with antibodies against Ki67 (SolA15), Bcl6 (7D1), Irf-4 (IRF4.3E4), Pax5 (1H9), Blimp-1 (5E7) purchased from Biolegend and Invitrogen and with c-Myc/n-Myc (D3N8F) from Cell Signaling Technology using the Thermo Fisher Scientific Foxp3/Transcription Factor Staining Buffer Set (00-5523-00). After surface staining, cells were fixed with fixation/permeabilization buffer and then further permeabilized according to the manufacturer's instructions. The intracellular staining antibody was added in permeabilization buffer for 60 min at RT.
Instrument	LSR Fortessa or FACSymphony™ A5 flow cytometer (BD Biosciences)
Software	FlowJo (versionS 9 & 10) was used for flow cytometry data analysis.
Cell population abundance	Purity of the sorted cells is checked periodically in our FACS facility and is generally equal or superior to 98 %.
Gating strategy	FSC-A/SSC-A plots were used to determine cell population gates excluding debris. FSC-H/FSC-W and SSC-SSW plots were then used to determine singlet gates. Additional gating was performed as described in main and supplementary figures.

☒ Tick this box to confirm that a figure exemplifying the gating strategy is provided in the Supplementary Information.
